# Functional interactions between NADPH oxidase 5 and actin

**DOI:** 10.3389/fcell.2023.1116833

**Published:** 2023-01-26

**Authors:** Samantha M. Richter, Lilyanna C. Massman, Dennis J. Stuehr, Elizabeth A. Sweeny

**Affiliations:** ^1^ Department of Biochemistry, The Medical College of Wisconsin, Milwaukee, WI, United States; ^2^ Department of Inflammation and Immunity, Lerner Research Institute, The Cleveland Clinic, Cleveland, OH, United States

**Keywords:** NOX5, actin, superoxide, cytoskeleton, ROS, PSN-1, calcium, reactive oxygen species

## Abstract

NADPH oxidase 5 (NOX5) is a transmembrane oxidative signaling enzyme which produces superoxide in response to intracellular calcium flux. Increasing evidence indicates that NOX5 is involved in a variety of physiological processes as well as human disease, however, details of NOX5 signaling pathways and targets of NOX5 mediated oxidative modifications remain poorly resolved. Actin dynamics have previously been shown to be modulated by oxidative modification, however, a direct connection to NOX5 expression and activity has not been fully explored. Here we show that NOX5 and actin interact in the cell, and each modulate the activity of the other. Using actin effector molecules jasplakinolide, cytochalasin D and latrunculin A, we show that changes in actin dynamics affect NOX5 superoxide production. In tandem, NOX5 oxidatively modifies actin, and shifts the ratio of filamentous to monomeric actin. Finally, we show that knockdown of NOX5 in the pancreatic cancer cell line PSN-1 impairs cell migration. Together our findings indicate an important link between actin dynamics and oxidative signaling through NOX5.

## 1 Introduction

NADPH oxidases (NOX/DUOX, EC 1.6.3) are a family of transmembrane enzymes that produce reactive oxygen species (ROS) (Bedard and Krause, 2007). There are seven family members, NOX1-5 and DUOX1&2. NOX5 is the most recently identified NOX/DUOX family member, and produces a superoxide burst in response to increases in intracellular calcium (Banfi et al., 2001; Cheng et al., 2001). While its physiological roles are still being uncovered, it has been shown to be critical for monocyte differentiation into dendritic cells (Marzaioli et al., 2017), differentiation and maturation of oligodendrocytes (Accetta et al., 2016), sperm motility and viability (Ghanbari et al., 2018), and vascular contraction (Montezano et al., 2018). Additionally, it has been implicated in cancers (Dho et al., 2015; Antony et al., 2017), diabetes (Holterman et al., 2014; Jha et al., 2017a), hypertension (Elbatreek et al., 2020; Camargo et al., 2022) and cardiovascular disorders (Santillo et al., 2015; Jha et al., 2017b). Due to the absence of NOX5 from mouse and rats, uncovering the mechanistic details regarding its role in the cell has lagged behind other NOX/DUOX family members.

As enzymes whose sole role is the production of ROS, it is no surprise that expression and activity of the NOX/DUOX family members are heavily regulated. NOX5 is no exception, and its activity is modulated by a growing list of factors, including peptide hormones (Montezano et al., 2010; Montezano et al., 2018), post-translational modifications (Jagnandan et al., 2007; Serrander et al., 2007; El Jamali et al., 2008; Pandey et al., 2011a; Pandey et al., 2011b; Qian et al., 2012; Chen et al., 2014b), protein:protein interactions (Chen et al., 2012; Chen et al., 2014a; Sweeny et al., 2020) and heme availability (Sweeny et al., 2020; Sweeny et al., 2021). How these factors, along with undiscovered players, intracellular calcium flux, and environmental stresses combine to drive either physiological processes or disease remains incompletely understood.

Reactive oxygen species (ROS) have been shown to regulate the actin cytoskeleton indirectly as well as through direct protein modifications. Oxidative modification of actin binding proteins including myosin II, gelsolin, cofilin, and L-plastin have been observed with important functional consequences (Xu et al., 2017; Balta et al., 2020). Additionally, proteins involved in actin regulation, including integrins, Rho family GTPases (Aghajanian et al., 2009), protein tyrosine phosphatases, Src family kinases (Camargo et al., 2022), focal adhesion kinase, protein kinase C, and calcium channels and transporters also undergo oxidative modifications which modulate their activity (Xu et al., 2017; Balta et al., 2020). Actin itself is also a target of oxidative modification. Oxidation of actin methionine residues by the protein Microtubule Associated Monooxygenase, Calponin And LIM Domain Containing 1 (MICAL1), triggers actin disassembly (Fremont et al., 2017; Grintsevich et al., 2017; Wioland et al., 2021) and has been implicated in crucial cellular functions including promoting F-actin clearance at the abscission site to allow completion of cytokinesis (Fremont et al., 2017). This oxidation is reversed by methionine-R-sulfoxide reductase B1 (MsrB1) allowing actin to repolymerize (Lee et al., 2013).

In fact, NOX enzymes and their co-factors have long been associated with the actin cytoskeleton. Treatment of granulocytes with cytochalasin B was found to decrease superoxide production in resting cells but increase superoxide production in cells exposed to phagocytic stimuli (Curnutte and Babior, 1975; Goldstein et al., 1975). Cytochalasin B inhibits phagocytosis in these cells, and therefore the increase in superoxide production was proposed to be due to increased localization to the plasma membrane (Curnutte and Babior, 1975; Goldstein et al., 1975; Root and Metcalf, 1977). The cytosolic factors p40^phox^, p47^phox^, and p67^phox^, required for NOX2 activation, have been shown to be associated with components of the cytoskeleton (Grogan et al., 1997; Wientjes et al., 2001; Touyz et al., 2005; Cave et al., 2006; Rasmussen et al., 2010; Shao et al., 2010) and this association appears to be important for spatial control of NOX2 activation (Cave et al., 2006; Rasmussen et al., 2010), its stimulation by angiotensin II (AngII) (Touyz et al., 2005), and actin organization (Grogan et al., 1997). Poldip2, a p22^phox^ binding partner (a transmembrane co-factor for NOX1-4), has been shown to work together with NOX4 to modulate stress fiber formation and cell migration (Lyle et al., 2009). Other studies have also shown that NOX4 interacts with components of the actin cytoskeleton (Hilenski et al., 2004), and that knockdown of NOX4 using shRNA prevents wound closure in 4T1 cancer cells (Zhang et al., 2013). The cytosolic co-factor for NOX2, p40^phox^, was found to be co-localized with filopodial actin bundles and co-localization with NOX2 and subsequent ROS production was observed when neurite growth was induced (Munnamalai et al., 2014), affecting F-actin content, retrograde F-actin flow and neurite outgrowth (Munnamalai et al., 2014). These associations expand beyond mammalian cells, as the yeast NOX, Yno1p, has also been shown to interact with actin and affect the actin cytoskeleton (Rinnerthaler et al., 2012; Weber et al., 2021), and NOX enzymes (NOX1 and NOX2) from the rice blast fungal pathogen are required for plant infection in processes involving F-actin remodeling (Ryder et al., 2013). Interestingly, despite the conserved requirement for ROS production, NOX1 and NOX2 mediate distinct processes both necessary for infection but crucially divergent from each other (Ryder et al., 2013).

NOX5 does not require p22^phox^, p40^phox^, p47^phox^, or Rac1 for activity, however, there are indications that it also interacts with the actin cytoskeleton. NOX5 overexpression in a brain microvascular endothelial cell line leads to phenotypic alterations including increased cell migration, and gene ontology analysis indicated changes to the cytoskeleton and Rho GTPase signaling (Marques et al., 2022). In human aortic endothelial cells thrombin stimulation induced NOX5 expression and ROS production, which led to cytoskeleton remodeling and cell migration (Pai et al., 2017). Finally, NOX5 has been shown to be a key player in human hypertension (Elbatreek et al., 2020; Camargo et al., 2022), and a recent paper has revealed that a component of vascular smooth muscle cell dysfunction is an AngII/NOX5/proto-oncogene tyrosine-protein kinase Src signaling network which can alter actin polymerization and cell migration (Camargo et al., 2022).

Overall, there are strong indications that the actin cytoskeleton contributes to controlling NOX activation, and that NOX activation leads to changes in the actin cytoskeleton. However, NOX5 does not rely on the co-factors implicated in actin mediated NOX regulation. Therefore, we sought to probe for a direct interaction between NOX5 and actin, and moreover, explore the effects of actin dynamics on NOX5 activity, and the effect of NOX5 activity on actin modification and polymerization state. Using the pancreatic cancer cell line PSN-1, which express endogenous NOX5, we were also able to show that NOX5 expression is correlated with cell migration, a process which relies on coordinated restructuring of the actin cytoskeleton. This work lays a foundation to further investigate the interactions and effects of these interactions between ROS producing NOX family members and components of the actin cytoskeleton and to understand the importance of these interactions in health and disease.

## 2 Materials and methods

For a full list of reagents and sources see Supplementary Materials.

### 2.1 Cell culture and transient transfection

There are six isoforms of NOX5, NOX5 -α, -β, -δ, -γ, -ε and -ζ. NOX5 -α and -β are the major isoforms and are catalytically active. The two appear to have similar activity and differ only by 18 amino acids which are at the N-terminus of NOX5α, but missing from NOX5β (Banfi et al., 2001; Pandey et al., 2012). In this study it is the NOX5β isoform which was transiently or stably expressed as described below.

HEK293 cells (human embryonic kidney cells immortalized by transfection with adenovirus 5, ATCC) and HEK293 cells stably expressing NOX5β (Banfi et al., 2004) were maintained in Dulbecco’s modified Eagle’s media (DMEM) with 10% fetal bovine serum (FBS) and antibiotic-antimycotic (Gibco, 1X, HEK293 cells) or G418 (Gibco, 400 μg/mL, HEK293 cells stably expressing NOX5β) at 37°C with 5% CO_2_ in a humidified incubator. PSN-1 cells (human pancreatic cancer cell line derived from adenocarcinoma (Yamada et al., 1986), ATCC) were maintained in RPMI 1640 (Cytiva) with 10% FBS and antibiotic-antimycotic (Gibco, 1X) in the same incubation conditions, with passaging facilitated with use of TrypLE (Gibco). For transfections, HEK293 cells were grown in DMEM with 10% FBS and no antibiotic. HEK293 cells were transfected with 5 μg DNA (empty vector, or NOX5β, Addgene) and 15 μL Lipofectamine 2000 (ThermoFisher Scientific) per 10 cm dish. Sixteen hours after transfection the media was changed to DMEM with 10% FBS and antibiotic-antimycotic. PSN-1 cells for transfection were grown in RPMI 1640 media with 10% FBS and no antibiotic and transfected with 50 nM of siRNA (non-targeting or NOX5 targeting, Dharmacon) and 25 μL of Lipofectamine 2000. Sixteen hours after transfection, the media was changed to RPMI 1640 with 10% FBS and antibiotic-antimycotic.

### 2.2 Western blot analysis

Cell lysates were normalized to total protein content as determined by a DC protein assay (Bio-Rad). SDS-PAGE and Western blot analysis were performed using standard procedures with antibodies against NOX5 (Proteintech) and β-actin (Sigma-Aldrich). Fluorescently labeled secondary antibodies (anti-rabbit and anti-mouse, LI-COR Biosciences) were used to visualize the proteins. Biotin was visualized using a fluorescently conjugated streptavidin (LI-COR Biosciences). Blots were imaged on a LI-COR Odyssey and analyzed using Empiria Studio 2.2 software (LI-COR Biosciences).

### 2.3 Co-immunoprecipitation assay

HEK293 and HEK293 cells stably expressing NOX5β were cultured as described above, then lysed with Cellytic M lysis buffer (Sigma-Aldrich) containing protease inhibitor (EDTA-free Protease Inhibitor Cocktail mini tablet, Sigma-Aldrich). Protein concentrations were determined by DC Protein Assay kit (Bio-Rad) per manufacturer’s instructions and 500 μg of protein was mixed with 2 μL mouse anti-β-actin primary antibody (Sigma-Aldrich) or 8 μL rabbit anti-NOX5 antibody (Proteintech), and PBS containing protease inhibitor (EDTA-free Protease Inhibitor Cocktail mini tablet, Sigma-Aldrich) for a total volume of 300 μL. Samples were incubated at 4°C while rotating for 2–3 h. After rotation, samples were added to a tube containing 50 μL of pre-washed Protein G Mag Sepharose beads (Cytiva) for the anti-β-actin antibody or Protein A Sepharose beads (Thermo Scientific) for the anti-NOX5 antibody. The sample and bead mixture was incubated at 4°C while rotating for 16 h. After incubation the supernatant was removed using a magnetic rack (Protein G Mag Sepharose beads) or centrifugation (Protein A Sepharose beads) and the beads washed 3 times with PBS (Sigma-Aldrich). 30 μL of 2x sample buffer (Bio-Rad) was added to each sample, boiled at 100°C for 5 min and subjected to SDS-page and western blot analysis as described above.

### 2.4 Proximity ligation assay with Duolink

Proximity Ligation Assays (PLA, Duolink, Sigma) for co-localization were performed according to the manufacturer’s protocol using primary antibodies to β-actin (Sigma-Aldrich 1:1000) and NOX5 (Proteintech, 1:1000), followed by a pair of oligonucleotide-labeled secondary antibodies (included in Duolink Kit). The assay detects positive signal only when the epitopes of the target proteins are in close proximity (<40 nm). The signal from each of the detected pair of PLA probes was then imaged using fluorescence microscopy (excitation/emission for Duolink red: 594/624; excitation/emission for DAPI: 360/460). One or both primary antibodies were omitted for negative controls.

### 2.5 Superoxide measurements

Cells were grown in phenol red free DMEM/F-12 media with 15 mM HEPES and L-glutamine with 10% FBS and 400 μg/mL G418 (or antibiotic-antimycotic, 1X, for HEK293 or PSN-1 cells). Cells were re-plated into clear bottom white tissue culture-treated 96-well plates (Costar) at a density of 2 × 10^5^ cells/well to achieve a confluency of 90% or greater at time of assay. L-012 (Wako Chemicals) at a concentration of 400 μM was added 60 min prior to read. The NOX5 superoxide burst was initiated by automated addition of 1 μM ionomycin (pre-treatment experiments, Figure 2, with luminescence read every 10 s for 300 s on a Flexstation3, Molecular Devices) or 0.5 μM ionomycin, 1 μM jasplakinolide (Bio-Techne), 1 μM cytochalasin D (Focus Biomolecules), or 1 μM latrunculin A (Focus Biomolecules), with luminescence read every 1 s for 180 or 300 s on a SpectraMax iD5 microplate reader (Figures 3, 5, Molecular Devices). Data were plotted and analyzed using GraphPad Prism v7.

### 2.6 Calcium flux measurements

Cells were grown in phenol red free DMEM/F-12 media with 15 mM HEPES and L-glutamine with 10% FBS and 400 μg/mL G418 (or antibiotic-antimycotic, 1X, for HEK293 control cells). Cells were re-plated into clear bottom black tissue culture-treated 96-well plates (Thermo Scientific) at a density to achieve a confluency of 90% or greater at time of assay. Cells were treated with FLIPR Calcium 6 Assay Kit (Molecular Devices) following manufacturer instructions. Per kit protocol, reagents were equilibrated to room temperature and loading buffer was made by fully dissolving component A into component B. Equal volume of loading buffer was then added directly to culture media (100 μL of buffer to 100 μL of culture media in each well). Plate was incubated at 37°C for 2 h. Measurement was performed on SpectraMax iD5 using SoftMax Pro Data Acquisition and Analysis Software (Molecular Devices).

### 2.7 Biotin switch assay

Cells were cultured and transfected as described above, then treated with 1 μM of ionomycin or DMSO control. Cells were lysed with Cellytic M lysis buffer (Sigma-Aldrich) containing protease inhibitor (EDTA-free Protease Inhibitor Cocktail mini tablet, Sigma-Aldrich) and 50 mM N-ethylmaleimide (Sigma-Aldrich). Protein concentrations were determined by DC Protein Assay kit (Bio-Rad) per manufacturer’s instructions. Equal concentrations of protein were precipitated with ice-cold acetone (Sigma-Aldrich), then reduced with tris(2-carboxyethyl)phosphine on resin (Thermo Scientific). Samples were then relabeled with 20 mM maleimide-PEG2-biotin (Thermo Scientific) for 24 h, and once again precipitated with ice-cold acetone. Samples were pulled down with streptavidin magnetic beads (New England BioLabs) for 24 h at a concentration of 1 mg of beads per 1 mg of protein and washed with and then suspended in ammonium bicarbonate (Sigma-Aldrich). The samples were analyzed using western blot analysis as described.

### 2.8 F-actin/G-actin assay

HEK293 cells (ATCC) were cultured at equal cell densities, transfected with NOX5β or empty vector DNA using methods described above, and lysed with F-Actin/G-Actin *In Vivo* Assay Biochem Kit (Cytoskeleton) according to the instructions provided (Alternatively, HEK293 cells (ATCC) and HEK293 cells stably expressing NOX5β were used). After lysing, the samples were homogenized using a 25G needle and incubated at 37°C for 10 min 100 μL of lysate were placed into 1.5 mL tubes and centrifuged at a speed of 350 × g at room temperature for 5 min. The supernatant was removed and placed into a labeled ultracentrifuge tube and centrifuged at 100,000 × g at 37°C for 1 h using Beckman-Coulter Optima MAX-TL Ultracentrifuge. Supernatant was gently removed and placed into labeled 1.5 mL tubes. Pellet was dissolved in 100 μL of F-actin depolymerization buffer (provided in kit) for 1 h on ice while pipetting up and down several times every 15 min. Samples were analyzed *via* western blot analysis as described above and band densities determined using Empiria Studio 2.2.

### 2.9 Scratch assay

PSN-1 cells were cultured and transfected with siRNA (non-targeting and NOX5-targeting, Dharmacon) as described above. Cells were then plated into clear 6, 12 or 96-well plastic plates (GenClone). Wound was made by scratching a vertical line through the cell monolayer using a 200 μL pipette tip (6 and 12-well plates) or by using a woundmaker (Essen Bioscience, 96-well plate). Immediately after wound creation, media was changed to fresh RPMI 1640 (Cytiva) with 10% FBS and antibiotic-antimycotic (Gibco, 1X). Data was collected using BioTek Cytation C10 for 18–20 h at 37°C and 5% CO_2_ and analyzed with Gen5 3.12 software.

### 2.10 Digital PCR

After completion of scratch assays PSN-1 cells were lysed in RLT buffer (Qiagen) and run through QIAshredder homogenizer columns (Qiagen). Lysates were then processed to extract mRNA (RNeasy kit, Qiagen) and converted to cDNA (High-capacity cDNA reverse transcription kit, Applied Bioscience). Equal concentrations of cDNA from PSN-1 cells transfected with non-targeting or NOX5-targeting siRNA were analyzed using a QIAcuity digital PCR system using the QIAcuity probe kit and the Taqman primer/probe assay kit for NOX5 from Thermo Scientific (Assay ID: Hs00225846_m1). Copies/µL of NOX5 transcript was normalized to the non-targeting siRNA control.

### 2.11 Quantification and statistical analysis

Statistical parameters including the exact value of n, the statistical test used to analyze the specific data set (two-tailed Student’s *t*-test or one-way ANOVA with appropriate post-test), and the parameters for statistical significance are reported in the Figures and Figure Legends. Data is presented as means ± SEM, and n represents biological replicates. Significance is denoted in the Figures with asterisks, and *p*-value cut-offs for the various statistical tests are listed in the Figure Legends (* denoting *p* < 0.05, **p < 0.01 and ***p < 0.001, for one-way ANOVA, and *p ≤ 0.05, **p < 0.001, ***p < 0.0001 for two-tailed *t*-test). Statistical analyses were performed with GraphPad Prism v7.

## 3 Results

**FIGURE 1:**
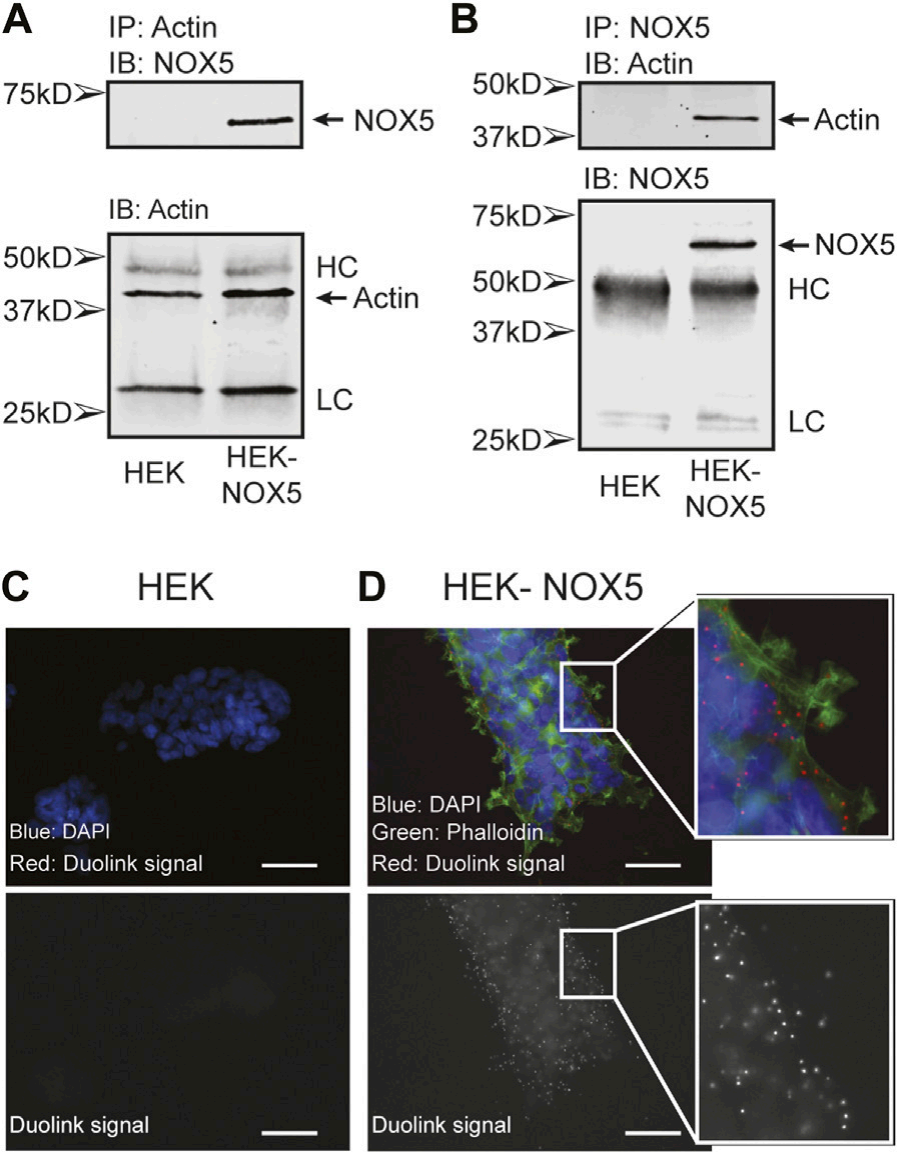
Actin and NOX5 interact in the cell. Co-immunoprecipitation of Actin and NOX5. Equal amounts of lysate from HEK293 and HEK293 cells stably expressing NOX5 were **(A)** incubated with anti-β-actin antibody and pulled down with Protein G Sepharose beads or **(B)** incubated with anti-NOX5 antibody and pulled down with Protein A Sepharose beads. After washing the precipitates were subjected to SDS-page and western blot analysis for β-actin and NOX5 (full blots in Supplementary Material, HC, heavy chain; LC, light chain). **(C,D)** The interaction between β-actin and NOX5 in HEK293 cells and HEK293 cells stably expressing NOX5 was probed using the Proximity Ligation Assay (PLA) Duolink kit from Sigma. Red foci represent interaction between the two proteins (<40 nm). **(C)** Control slide of HEK293 cells with both primary antibodies (anti-NOX5 and anti-β-actin) and both probes. Top panel shows overlap of DAPI (blue) and Duolink signal (red). Bottom panel shows red channel signal in greyscale. In the absence of NOX5 expression, the assay does not result in red foci. **(D)** Addition of both antibodies and probes to the HEK293 cells stably expressing NOX5 results in bright red foci indicating an interaction. Top panel shows overlap of DAPI (blue), phalloidin for filamentous actin (green) and Duolink signal (red). Bottom panel shows red channel signal in greyscale. Scale bars are 50 µm.

Actin and NOX5 interact in the cell. To probe a possible interaction between actin and NOX5 in the cell, we performed co-immunoprecipitation assays and the proximity ligation assay Duolink (Sigma). Co-immunoprecipitation studies using HEK293 cells and HEK293 cells stably expressing NOX5 showed that actin and NOX5 can each pull the other down in NOX5 expressing cells (Figures 1A, B). To confirm this interaction further, we used the proximity ligation assay Duolink, which results in red foci when two target proteins (NOX5 and β-actin) are in close proximity (<40 nm). Performing this assay in HEK293 cells (Figure 1C), or in HEK293 cells that stably express NOX5, but with only one of the two primary antibodies added (either anti-NOX5 or anti-β-actin, Supplementary Figure S1) resulted in no visible foci, while in HEK293 cells stably expressing NOX5 with both primary antibodies added the assay resulted in bright red foci indicating the proteins were in close proximity (Figure 1D).

Pre-treatment of cells with compounds affecting the actin cytoskeleton modulates NOX5 superoxide production upon calcium stimulation. To assess the role of actin dynamics and its downstream effects on NOX5 superoxide production, we pre-treated HEK293 cells and HEK293 cells stably expressing NOX5 with actin effector compounds prior to triggering a NOX5 superoxide burst with ionomycin calcium. Calcium binding to the N-terminal EF hands of NOX5 triggers an electron transfer chain culminating in the reduction of dioxygen to superoxide. Therefore, a superoxide burst by NOX5 can be stimulated in cells by the addition of ionomycin calcium salt and measured using the superoxide sensor L-012 (Daiber et al., 2004; Qian et al., 2012; Sweeny et al., 2020). We measured the calcium-sensitive superoxide burst in cells pre-treated for 10, 30, 60 or 120 min with vehicle control (DMSO), and either low or high concentrations of jasplakinolide (0.2 or 2 μM) cytochalasin D (0.5 or 2 μM), or latrunculin A (0.5 or 5 μM). Protein translation was inhibited with cycloheximide before cell treatment to eliminate contributions by newly synthesized NOX5. Jasplakinolide is a cell-permeable cyclic peptide which is a potent inducer of actin polymerization (Bubb et al., 1994) and cytochalasin D and latrunculin A are inhibitors of actin polymerization (Casella et al., 1981; Schliwa, 1982; Hayot et al., 2006; Fujiwara et al., 2018) through different mechanisms. Cytochalasin D has been reported to bind the barbed end of actin (Hayot et al., 2006), blocking growth of actin filaments, and may primarily affect F-actin that results from rapid polymerization induced by stimuli (Casella et al., 1981). Additionally, treatment with cytochalasin D can lead to the formation of filamentous aggregates, or foci (Schliwa, 1982). Latrunculin A blocks actin polymerization by binding and sequestering actin monomers as well as promoting dissociation of actin filaments (Fujiwara et al., 2018). We found that, as expected, treatment with jasplakinolide increased the F/G actin ratio while latrunculin A decreased the F/G actin ratio (Supplementary Figure S2). However, in our hands, cytochalasin D did not decrease the F/G ratio (Supplementary Figure S2), likely due to its ability to promote actin aggregates which may pellet with the filamentous actin in our assay. This is supported by immunofluorescence imaging of HEK293 cells and HEK293 cells stably expressing NOX5 in which we see that cytochalasin D treatment results in disrupted actin filaments and large actin aggregates, while latrunculin A treatment results in near elimination of actin filaments with only stringy remnants and small foci (Supplementary Figure S3).

**FIGURE 2:**
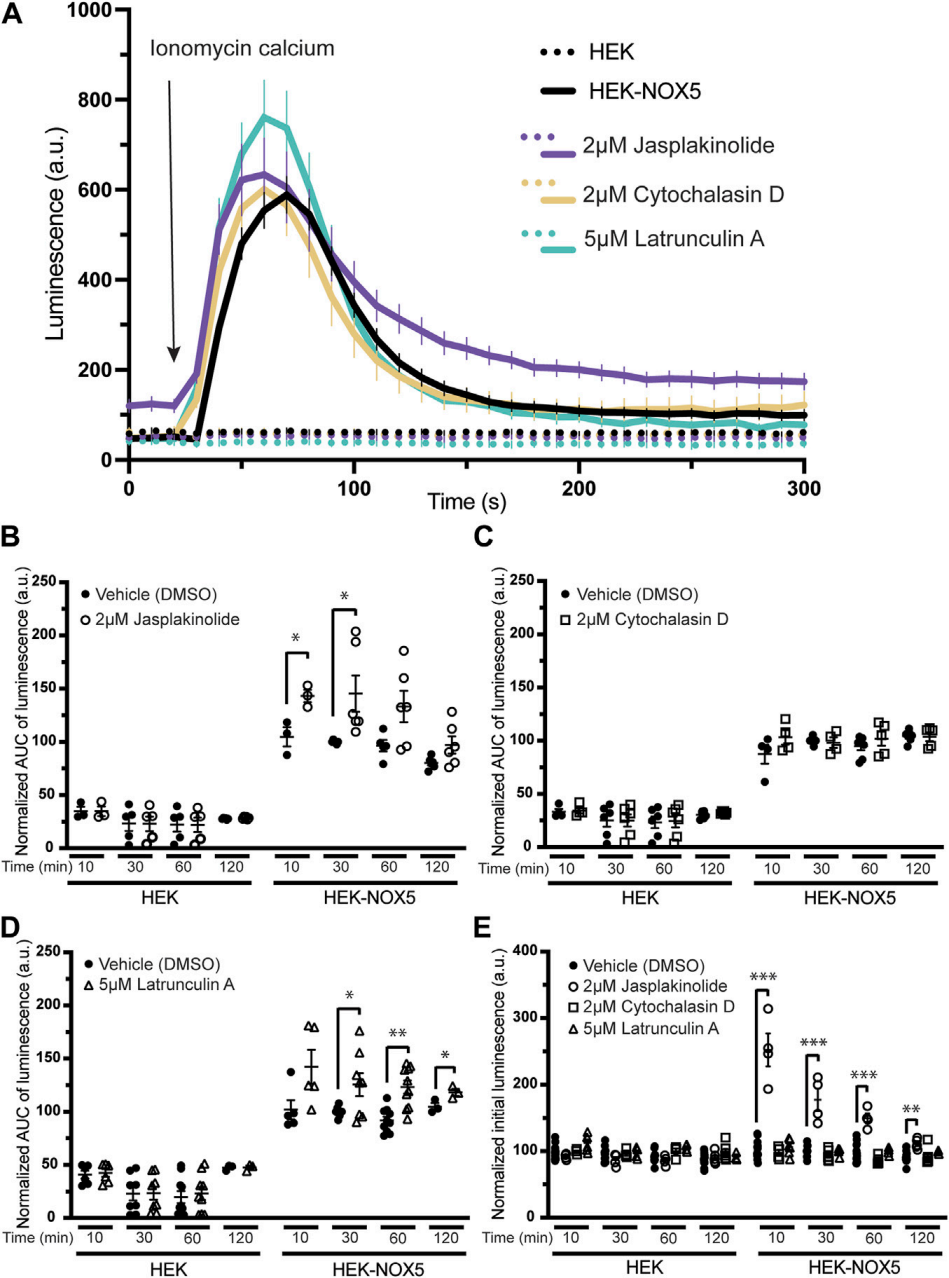
Molecules affecting actin dynamics modulate NOX5 superoxide production upon calcium stimulation. Superoxide levels were detected using the luminol analog L-012, which luminesces in the presence of superoxide. Before any incubation with actin effector molecules, the cells were treated with cycloheximide to block protein translation. Addition of ionomycin calcium salt triggers a NOX5 superoxide burst. **(A)** Timecourse of superoxide production by HEK293 cells and HEK293 cells stably expressing NOX5 pre-treated for 30 min with vehicle control (DMSO), jasplakinolide (2 μM), cytochalasin D (2 μM) or latrunculin A (5 μM). **(B)** Quantification of NOX5 superoxide production (area under the curve) after treatment for 10, 30, 60 or 120 min with DMSO or **(B)** jasplakinolide (2 μM), **(C)** cytochalasin D (2 μM) or **(D)** latrunculin A (5 μM). Values represent means ± SEM, *n* = 3–10. Differences in NOX5 activity with treatment vs. DMSO control were assessed using Student’s *t*-test with * denoting *p* < 0.05, and **p < 0.001. **(E)** Unstimulated superoxide production by NOX5 after treatment for 10, 30, 60 or 120 min with DMSO, jasplakinolide (2 μM), cytochalasin D (2 μM) or latrunculin A (5 μM). Values represent means ± SEM, *n* = 3–10. Differences in basal NOX5 activity at each timepoint after treatment with actin affecting molecules vs. DMSO control were assessed using a one-way ANOVA with Dunnett’s test with ** denoting *p* < 0.01 and ***p < 0.001.

Figure 2A shows a timecourse of superoxide production upon automated addition of ionomycin calcium after a 30 min pre-treatment with DMSO or the high concentration of actin effector. Pre-treatment with the compounds did not affect calcium flux induced by the addition of ionomycin Supplementary Figure S4A). HEK293 cells, which do not express NOX5, did not produce measurable superoxide in any treatment group. Pre-treatment with jasplakinolide resulted in slightly elevated peak superoxide production and the signal remained elevated to the end of the experiment. Pre-treatment with cytochalasin D appeared to have little to no effect, while treatment with latrunculin A resulted in an elevated peak. Unlike jasplakinolide, pre-treatment with latrunculin A did not result in an elevated signal after resolution of the superoxide burst. A 30 min pre-treatment with the lower concentrations of the compounds had similar effects on NOX5 superoxide production; pre-treatment with jasplakinolide and latrunculin A resulted in elevated superoxide production, while pre-treatment with cytochalasin D had no effect (Supplementary Figure S4B). Again, jasplakinolide treatment alone resulted in a slightly elevated signal independent of the superoxide burst, however, it was less pronounced at the lower concentration. Total superoxide production (area under the curve) for each high concentration treatment group are shown in Figure 2B (jasplakinolide) Figure 2C (cytochalasin D) and Figure 2D (latrunculin A). Again, pre-treatment with cytochalasin D did not show an effect on NOX5 superoxide production at any timepoint measured, while jasplakinolide and latrunculin A pre-treatment enhanced NOX5 superoxide production.

During the course of these assays, we observed that pre-treatment with jasplakinolide also increased the baseline luminescence of cells stably expressing NOX5 (Figure 2E), indicating that this compound may be able to stimulate NOX5 basal activity at endogenous cytosolic calcium levels. This increase in basal activity was not seen after pre-treatment with cytochalasin D or latrunculin A and was not observed for the HEK293 cells which do not express NOX5. The lower concentration of jasplakinolide also resulted in elevated basal activity but to a smaller degree, indicating it may be a concentration dependent effect (Supplementary Figure S4C).

**FIGURE 3:**
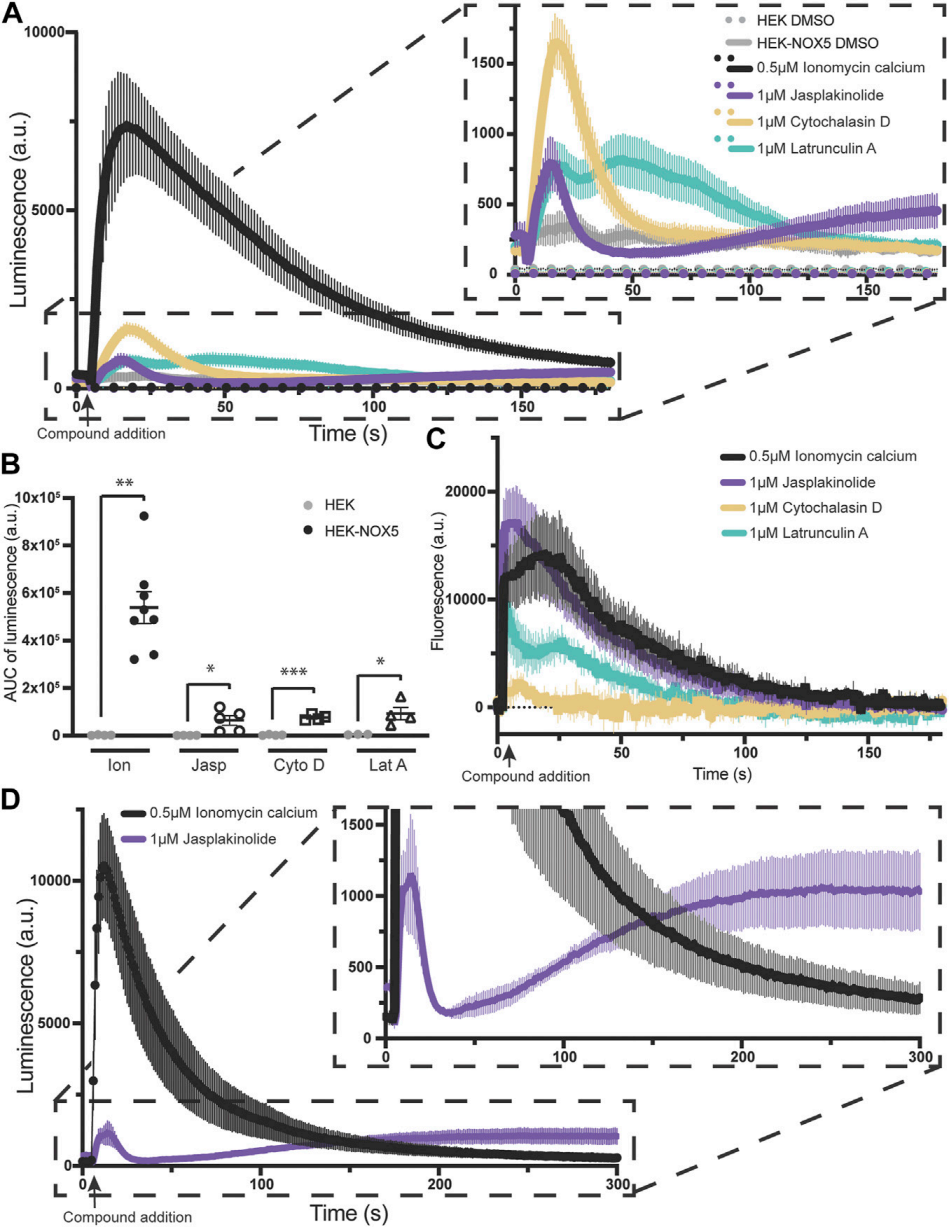
Actin effectors alone are sufficient for NOX5 stimulation. **(A)** Timecourse of superoxide production by HEK293 cells and HEK293 cells stably expressing NOX5 after automated addition of DMSO, ionomycin calcium (0.5 μM), jasplakinolide (1 μM), cytochalasin D (1 μM) or latrunculin A (1 μM) and measured using L-012 luminescence. **(B)** Quantification of NOX5 superoxide production (area under the curve). Values represent means ± SEM, *n* = 3–8. Differences in superoxide production upon compound addition by HEK293 cells stably expressing NOX5 compared to HEK293 control cells were assessed using Student’s *t*-test with * denoting *p* < 0.05, **p < 0.001 and ***p < 0.0001. **(C)** Changes in intracellular calcium levels after automated addition of DMSO, ionomycin calcium (0.5 μM), jasplakinolide (1 μM), cytochalasin D (1 μM) or latrunculin A (1 μM) measured using the FLIPR Calcium 6 Assay Kit (Molecular Devices) and normalized to baseline fluorescent signal. **(D)** Extended timecourse of superoxide production by HEK293 cells stably expressing NOX5 after automated addition of ionomycin calcium (0.5 μM) or jasplakinolide (1 μM) and measured using L-012 luminescence.

Superoxide production by NOX5 can be triggered by actin effectors alone in a manner partially independent of the effect on intracellular calcium flux. Due to the elevated baseline after pre-treatment with jasplakinolide, we sought to determine the effect of the actin effector compounds on NOX5 activity in the absence of ionomyocin calcium addition. As the two concentrations used previously displayed similar effects, we simplified our protocol and used one concentration for each compound. Therefore, we compared superoxide production by NOX5 after automated addition of vehicle control (DMSO), ionomycin calcium (0.5 μM, positive control), or 1 μM of either jasplakinolide, cytochalasin D or latrunculin A. All three actin effectors elicited a superoxide burst upon addition (Figure 3A, quantification of area under the curve shown in Figure 3B). Surprisingly, despite showing no effect with pre-treatment, cytochalasin D elicited the largest peak in superoxide production. To determine whether the stimulation of NOX5 superoxide production by the actin effector molecules was due to changes in available intracellular calcium we employed an intracellular calcium sensor which increases in fluorescence upon calcium binding. We found that addition of all three actin effectors caused some amount of increase in intracellular calcium (Figure 3C). However, the magnitude of the increases in calcium did not always match the relative magnitude of NOX5 superoxide production. As expected, ionomycin calcium resulted in a robust increase in fluorescence indicating increased intracellular calcium. This matched the luminescence data indicating a robust stimulation of NOX5 superoxide production. Jasplakinolide also elicited a large change in fluorescence indicating increased intracellular calcium, however, the initial superoxide burst stimulated by jasplakinolide was minimal compared to that elicited by ionomycin calcium and only approximately 50% of that elicited by cytochalasin D despite that compound causing only a minimal change in calcium levels (∼10% of the calcium increase upon addition vs. the effect of jasplakinolide, Figure 3C). This indicates that the superoxide burst caused by cytochalasin D can only be partially explained by increased intracellular calcium. Interestingly, a second phase of NOX5 superoxide production appears to be stimulated by jasplakinolide (Figures 3A, D for a longer timecourse) which appears to be independent of changes in calcium levels, as the signal from the calcium sensor has returned to baseline by 180s (Figure 3C). Latrunculin A induced changes in calcium levels and stimulation of NOX5 superoxide production appear to match in terms of their bimodal nature (Figures 3A, C), however, the cause of the bimodality is unclear. Interestingly, the signal from the calcium sensor in HEK293 cells not expressing NOX5 does not display a bimodal calcium response to latrunculin A (Supplementary Figure S5) indicating there may be an interplay between NOX5 and latrunculin A mediated calcium increases.

**FIGURE 4:**
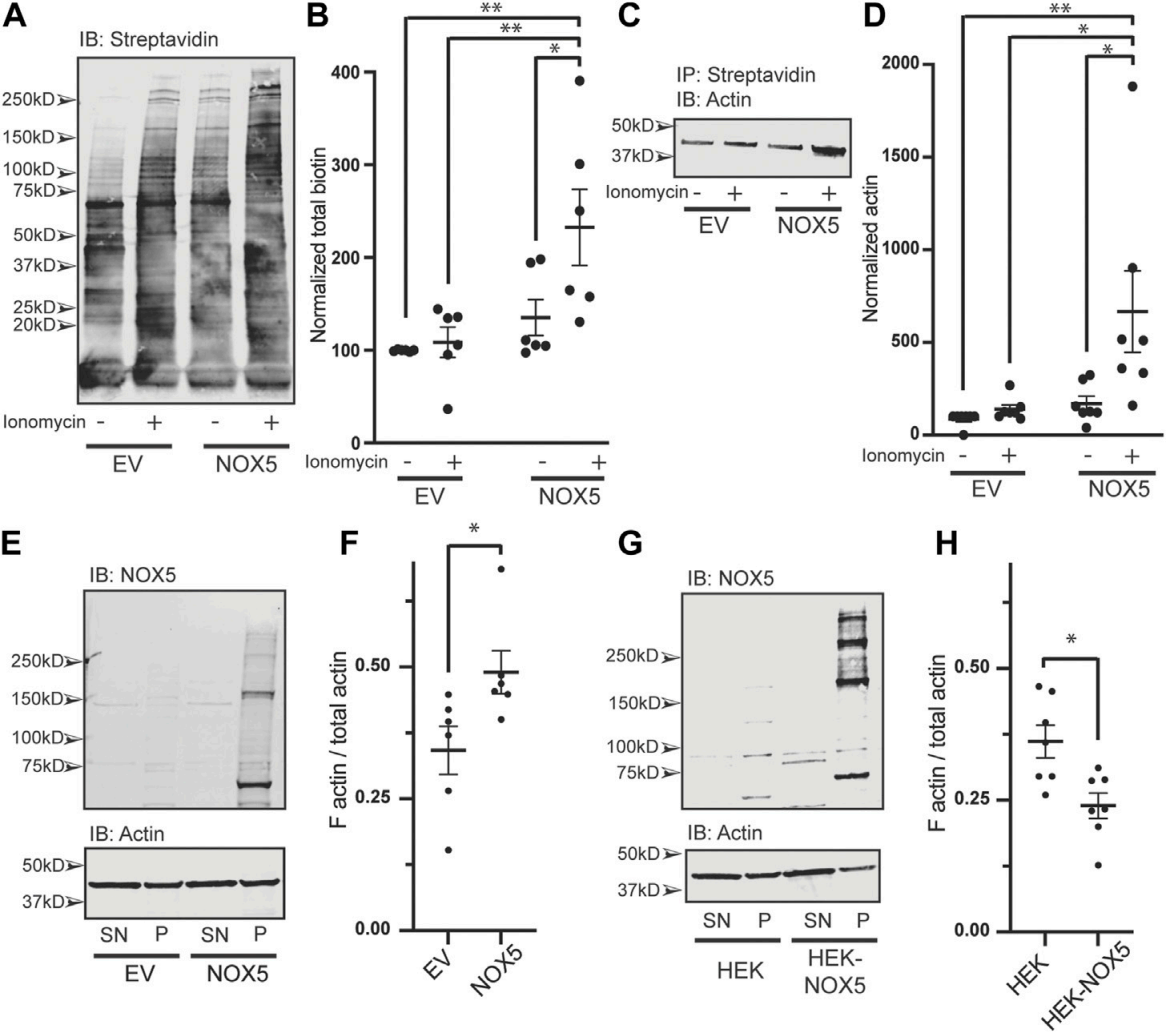
NOX5 expression and activity enhances oxidative modification of actin and shifts the F/G actin ratio in the cell. HEK293 cells were transiently transfected with empty vector (EV) or NOX5 and then treated with DMSO control or 1 μM ionomycin calcium for 5 min. After treatment cells were lysed and subjected to a biotin switch assay to label cysteine residues which had been reversibly oxidized in the cell. Biotinylated proteins were pulled out using streptavidin beads and analyzed by western blot. **(A)** Representative western blot showing biotin labeling in transfected cells with quantification shown in **(B)**. Values represent means ± SEM, *n* = 6. Differences in total biotin labeling between the samples was assessed using a one-way ANOVA with Tukey’s test with * denoting *p* < 0.05 and **p < 0.01. **(C)** Representative western blot of biotin switch samples co-immunoprecipitated using streptavidin beads and then blotted for β-actin. **(D)** Quantification of biotin labeled β-actin with values representing means ± SEM, *n* = 7. Differences in biotin labeled actin between the samples was assessed using a one-way ANOVA with Tukey’s test with * denoting *p* < 0.05 and **p < 0.01. The filamentous to free globular actin ratio (F/G) was determined using the F-Actin/G-Actin *In Vivo* Assay Biochem Kit (Cytoskeleton) for transiently transfected HEK293 cells [EV vs. NOX5, **(E,F)**] and HEK293 cells vs. HEK293 cells stably expressing NOX5 **(G,H)**. Representative western blots of actin and NOX5 found in the supernatant (SN) vs. pellet (P) are shown in **(E)** (transient transfectants) and **(G)** (stable expression) with quantification in **(F)** (transient transfectants, values represent means ± SEM, *n* = 6) and **(H)** (stable expression, values represent means ± SEM, *n* = 7). Differences in the F/G actin ratio were assessed using Student’s *t*-test with * denoting *p* < 0.05. Full blots can be found in Supplementary Material.

NOX5 expression and activity results in oxidative modification of actin and changes to the F/G actin ratio. We found that chemically mediated changes in actin dynamics can stimulate superoxide production by NOX5 (Figures 2, 3; Supplementary Figure S4). However, we were also interested in understanding the effect of NOX5 on actin. We employed a biotin switch assay which adds a biotin tag to cysteine residues which are reversibly oxidized. These samples were then subjected to co-immunoprecipitation with streptavidin beads to pull-down the biotin labeled proteins and subjected to western blot analysis and probed for biotin (Figure 4A, quantification in Figure 4B) and actin (Figure 4C, quantification in Figure 4D). After treatment with ionomycin calcium, HEK293 cells transiently transfected with empty vector displayed a slight increase in total biotin labeling of proteins (Figure 4A, quantification in Figure 4B) and biotin labeled actin (Figure 4D), however, these changes were not statistically significant. Treatment with ionomycin calcium of HEK293 cells which were transiently transfected with NOX5 resulted in a significant increase in total biotin labeling of proteins (Figure 4A, quantification in Figure 4B) and in the amount of biotin labeled actin (Figure 4D), indicating that NOX5 superoxide production leads to increases in oxidative modification of actin. This was also true for the HEK293 cells stably expressing NOX5 (Supplementary Figure S6A), although the effect was more variable, possibly due to the contribution of irreversible oxidative modifications which are not detected by our biotin switch assay. We also assessed whether NOX5 expression influenced the F/G actin ratio. Surprisingly, transient transfection of HEK293 cells with NOX5 had the opposite effect on the F/G actin ratio compared to HEK293 cells stably expressing NOX5 (Figures 4E–H). Transient transfection of NOX5 resulted in an increase in the F/G actin ratio (Figure 4E, quantification in Figure 4F), while comparison of HEK293 cells to HEK293 cells stably expressing NOX5 indicate a decrease in the F/G actin ratio in the cells expressing NOX5 (Figure 4G, quantification in Figure 4H). Together this indicates that NOX5 expression may have differential effects on the actin cytoskeleton based on expression level and/or duration, as these differ between transient and stable transfections with higher and prolonged expression in the HEK293 cells stably expressing NOX5 compared to the HEK293 cells transiently expressing NOX5 (Supplementary Figures S6B, C).

**FIGURE 5:**
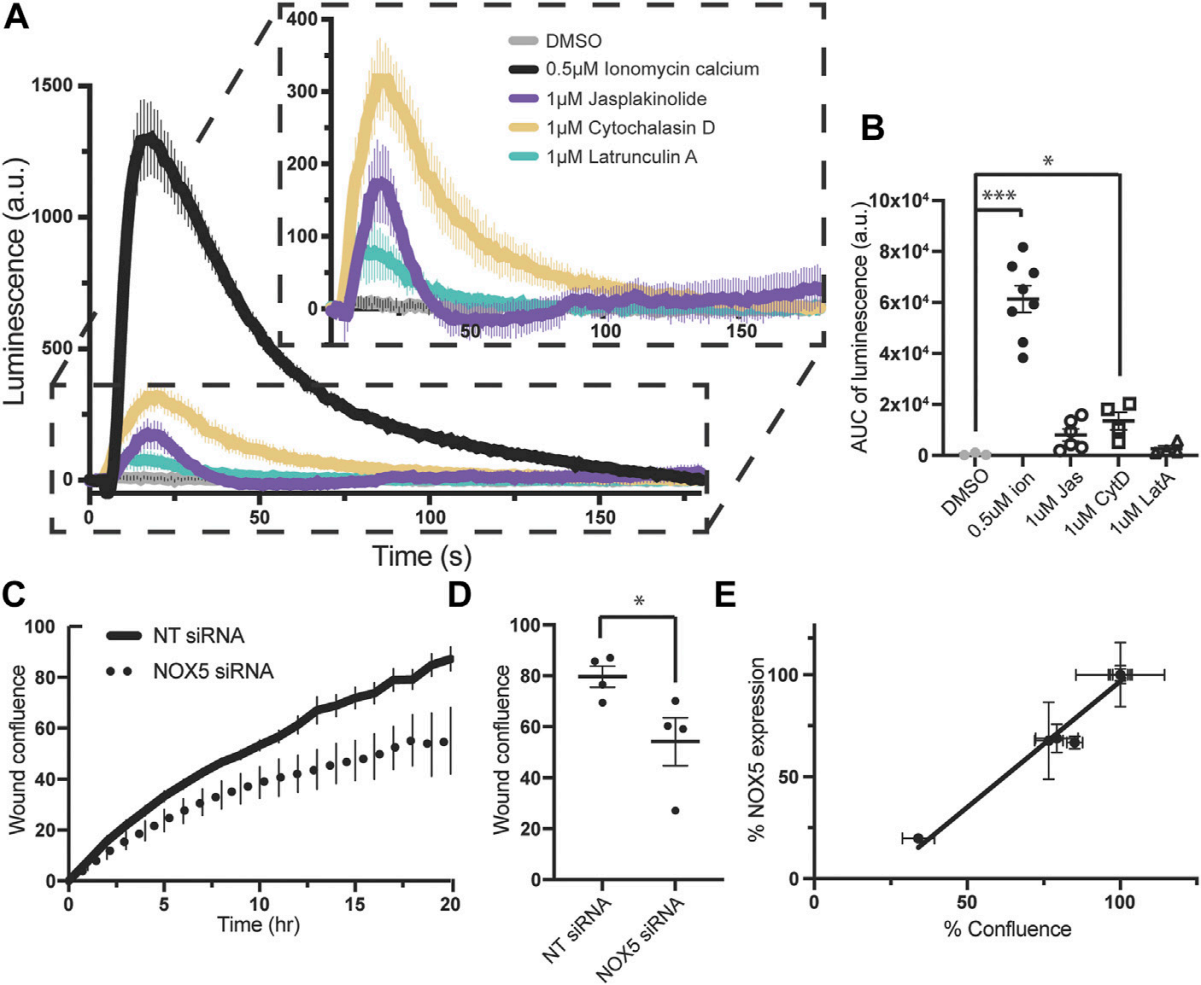
Superoxide production by endogenously expressed NOX5 in PSN-1 cells is stimulated by actin effector molecules and NOX5 knockdown by siRNA decreases PSN-1 cell migration. **(A)** Timecourse of superoxide production by PSN-1 cells after automated addition of DMSO, ionomycin calcium (0.5 μM), jasplakinolide (1 μM), cytochalasin D (1 μM) or latrunculin A (1 μM) and measured using L-012 luminescence. **(B)** Quantification of NOX5 superoxide production (area under the curve). Values represent means ± SEM, *n* = 3–8. Differences in superoxide production upon compound addition compared to DMSO control were assessed using Student’s *t*-test with * denoting *p* < 0.05, and ***p < 0.0001. PSN-1 cells were transfected with non-targeting (NT) or NOX5 targeting siRNA and subjected to a scratch assay to measure cell migration. **(C)** Timecourse of wound confluence after scratch with quantification of wound confluence at 18 h shown in **(D)**. Values represent means ± SEM, *n* = 4. **(E)** Efficiency of NOX5 knockdown by siRNA (NOX5 expression after transfection with NOX5 siRNA normalized to NT siRNA) plotted against % confluence at assay completion. Error bars reflect the technical replicates of each experiment with *n* = 2–3 for each.

Superoxide production by endogenously expressed NOX5 is stimulated by actin effector molecules, and knockdown of NOX5 impairs cell migration. To assess whether these NOX5:actin effects occurred with endogenously expressed NOX5, we employed PSN-1 cells, a human pancreatic adenocarcinoma cell line in which NOX5 is among the highest over-expressed genes (Rouillard et al., 2016). We found that addition of ionomycin calcium, jasplakinolide, cytochalasin D or latrunculin A resulted in similar patterns of superoxide production in PSN-1 cells (Figures 5A, B) compared to our HEK293 cells stably expressing NOX5 (Figure 3A), indicating that the effect of actin dynamics on NOX5 superoxide production is physiologically relevant.

We then used siRNA to knockdown NOX5 in these cells and assessed the effect on cell migration. Cell migration relies on coordinated and dynamic assembly and disassembly of actin filaments and increasing evidence supports a central role for ROS, especially derived from NOX enzymes, in this process (Qian et al., 2005; Boudreau et al., 2012; Zhang et al., 2013; Alam et al., 2014; Pai et al., 2017; Xu et al., 2017; Balta et al., 2020; Camargo et al., 2022; Marques et al., 2022). Recent work has specifically implicated NOX5 in cell migration in colon cancer cell lines (Ashizawa et al., 2021), coronary smooth muscle cells (Gole et al., 2014), and human brain microvascular endothelial cells (Marques et al., 2022). Therefore, we employed a scratch assay in which images were taken every hour for 18–20 h and assessed for scratch wound confluence. We found that knockdown of NOX5 in PSN-1 cells impaired cell migration as assessed by the wound confluence timecourse (Figure 5C) and the wound confluence at 18 h (Figure 5D). Moreover, the decrease in wound confluence was highly correlated with the effectiveness of NOX5 knockdown (i.e., decreasing NOX5 expression results in decreased cell migration, Figure 5E).

## 4 Discussion

Rapid actin assembly and disassembly are crucial for a wide array of cellular processes and dysregulation of these dynamics can result in human disease. Therefore, the actin cytoskeleton is heavily regulated by direct actin binding partners and numerous signaling cascades. Entwined in this complex network of factors are oxidative modifications mediated by ROS. A main source of ROS are the NOX enzymes, and indeed there is evidence of these enzymes interacting with the actin cytoskeleton and modulating its behavior (Grogan et al., 1997; Hilenski et al., 2004; Touyz et al., 2005; Cave et al., 2006; Lyle et al., 2009; Rinnerthaler et al., 2012; Ryder et al., 2013; Zhang et al., 2013; Munnamalai et al., 2014; Weber et al., 2021). Importantly, these interactions appear to have physiological consequences. Cell migration, a process in which multiple NOX family members have been implicated (Lyle et al., 2009; Boudreau et al., 2012; Alam et al., 2014; Pai et al., 2017; Camargo et al., 2022; Marques et al., 2022), is essential for the process of metastasis and relies on large scale remodeling of the actin cytoskeleton (Skonieczna et al., 2017; Breitenbach et al., 2018). Additionally, there is mounting evidence that ROS produced by NOX enzymes contribute to vascular dysfunction through actin remodeling (Xu et al., 2017; Touyz et al., 2018; Camargo et al., 2022). Here we show that the calcium responsive NOX5 directly interacts with actin, and that this interaction has functional consequences both for NOX5 superoxide production as well as actin dynamics and cell migration of the pancreatic cancer cell line PSN-1.

In this study we used three compounds with robust but divergent effects on the actin cytoskeleton to assess how these changes affected the activity of NOX5. Surprisingly, the compounds had differential effects depending on whether they were used as a pre-treatment before NOX5 stimulation with ionomycin calcium or added directly at endogenous cytosolic calcium levels. Additionally, while all three elicited some amount of increase in intracellular calcium, it was not sufficient to explain the effect on NOX5 activity. Pre-treatment of NOX5 expressing cells with jasplakinolide, a compound which stabilizes filamentous actin, led to increased basal (non-stimulated) superoxide production by NOX5, and increased superoxide production upon stimulation with calcium. Pre-treatment with latrunculin A, which promotes F-actin disassembly, also enhanced superoxide production upon calcium stimulation, but did not display any effects on basal NOX5 activity. Importantly, the effects of pre-treatment with jasplakinolide and latrunculin A on NOX5 superoxide production appear to be independent of their effects on intracellular calcium levels, as calcium measured using an intracellular sensor had returned to baseline before the first pre-treatment timepoint (calcium returns to baseline within 3 min after compound addition, and the first pre-treatment timepoint is 10 min after addition), and pre-treatment with these compounds did not affect the calcium flux induced by ionomycin calcium addition. Pre-treatment with cytochalasin D, a compound which can inhibit actin filament assembly and promote actin aggregates, had no effect on ionomycin calcium stimulated NOX5 superoxide production, however, direct addition of the compound resulted in the most pronounced superoxide burst from NOX5 observed for the three compounds despite eliciting a barely detectable spike in intracellular calcium. Whether the minimal change in calcium could be sufficient for the NOX5 superoxide production observed is unknown, however, the timing of the cytochalasin D induced increase in intracellular calcium and NOX5 superoxide production overlaps in a manner suggesting it may be possible. Indeed, it is feasible that a small, localized increase in calcium could elicit a larger NOX5 superoxide burst than a larger and more diffuse increase in calcium. Additionally, with this system we were unable to differentiate between calcium influx from the media or calcium release from intracellular stores. Whether one or both are responsible for the observed increases in intracellular calcium may influence the spatial distribution of calcium in the cell and the degree to which a given intracellular concentration of calcium may be able to stimulate NOX5 activity. These questions can now be explored using higher resolution *in vivo* calcium measurements.

Direct addition of jasplakinolide and latrunculin A also induced NOX5 superoxide production. Surprisingly, jasplakinolide, which had the largest effect with pre-treatment, and the largest increase in intracellular calcium, elicited the smallest stimulation of NOX5 superoxide production. However, there appears to be a secondary mode of NOX5 stimulation after the initial superoxide burst that more closely resembles NOX5 stimulation by phorbol 12-myristate 13-acetate (PMA) (Jagnandan et al., 2007). Treatment with PMA results in NOX5 phosphorylation, which sensitizes the enzyme to calcium and results in activation at endogenous calcium levels (Jagnandan et al., 2007). These results suggest that treatment with jasplakinolide may elicit transient or prolonged changes in NOX5 phosphorylation. This can now be investigated as well. Latrunculin A displayed a bimodal effect on both NOX5 superoxide production and intracellular calcium. This bimodal calcium release was not seen in HEK293 cells not expressing NOX5, suggesting that the initial superoxide burst stimulated by latrunculin A may lead to a NOX5 dependent increase in cytosolic calcium leading to a feed-forward mechanism of continued activation, similar to its role in vascular smooth muscle cell phenotypic switching and calcification (Furmanik et al., 2020). Further work can now address how the actin cytoskeleton may be modulating the effect of NOX5 on intracellular calcium levels and how this is related to the recently reported calcium-mediated actin reset, in which increased intracellular calcium causes rapid large scale changes in the actin cytoskeleton (Wales et al., 2016).

Other factors which may be contributing to the changes in NOX5 activity elicited by the compounds include changes in NOX5 localization and/or oligomerization. NOX5 primarily resides in the ER membrane but has been observed to translocate to the plasma membrane upon stimulation. Since the superoxide sensor measures extracellular superoxide, increased translocation of NOX5 to the plasma membrane would lead to increased detection of superoxide, reminiscent of the effect of cytochalasin B on superoxide production by stimulated granulocytes (Curnutte and Babior, 1975; Goldstein et al., 1975; Root and Metcalf, 1977). However, NOX5 does not rely on the cytosolic factors involved in the changes in NOX2 localization upon actin cytoskeleton perturbation (Cave et al., 2006; Rasmussen et al., 2010). Therefore, changes in the localization of NOX5 in the resting or activated state should be further investigated, and if these changes do occur it is likely through different mechanisms. Our immunofluorescent imaging experiments (Supplementary Figure S3) did not reveal large scale changes in NOX5 localization; however, these were in unstimulated conditions, and lacked the appropriate co-localization markers to determine whether changes occur. Further work using quantitative co-localization imaging techniques and cellular fractionation studies will shed light on the question of NOX5 localization in response to these compounds. Additionally, it has been shown that changes in NOX5 oligomerization influences stimulated superoxide production (Sweeny et al., 2020). Whether these compounds affect the rate of formation, size, or conformation of NOX5 oligomers remains to be seen, and may have important consequences for its activity.

This study also showed that NOX5 expression and activity leads to increased reversible oxidative modification of actin and that NOX5 expression effects the F/G actin ratio in cells. Importantly, this work supports the previously observed dichotomy of the effect of ROS on F-actin. In some cases, especially in the presence of high concentrations of oxidants, actin oxidation leads to decreases in F-actin (Xu et al., 2017; Balta et al., 2020) and this is clearly the case for the methionine oxidizing enzyme MICAL1 (Fremont et al., 2017; Grintsevich et al., 2017; Wioland et al., 2021). However, other work has found that lower levels of ROS can instead promote F-actin assembly and formation of stress fibers (Aghajanian et al., 2009; Lyle et al., 2009; Munnamalai et al., 2014; Xu et al., 2017; Balta et al., 2020; Camargo et al., 2022). Here we show that the effect of NOX5 on the F/G actin ratio depends upon whether expression is transient or stable, indicating that changes in expression and activation of NOX5 can have opposing effects on actin dynamics. One possibility is that prolonged overexpression of NOX5 leads to more irreversible modifications, and that these modifications lead to different outcomes for the actin cytoskeleton. Future work correlating the nature of the modification to its effect will be a crucial point in developing our understanding of the roles of NOX5 in human health and disease.

Finally, we showed that the effects of actin modulation on NOX5 superoxide production is conserved in the cancer cell line PSN-1 which expresses high levels of endogenous NOX5, and we found that knockdown of NOX5 using siRNA in these cells decreased cell migration to a degree correlated with the effectiveness of the knockdown. This indicates that our findings are relevant to physiological and disease states involving the expression and activity of NOX5.

This and previous studies have indicated that a myriad of factors determine the outcome of ROS mediated effects on actin dynamics and many important questions remain. Future work can now investigate the role of other accessory proteins in the NOX5:actin interaction, as well as determine whether specific antioxidant proteins are responsible for reversing the NOX5 mediated modification of actin. While we have shown that NOX5 and actin directly interact in the cell, a direct interaction is not necessarily required for NOX5 to mediate oxidative modification of actin. Additional factors are no doubt involved in the NOX5/calcium/actin network, and understanding how they coordinate, as well as how the composition and effects change by cell type and disease state will be crucial for a broad understanding of NOX mediated ROS effects on the actin cytoskeleton.

## Data Availability

The raw data supporting the conclusion of this article will be made available by the authors, without undue reservation.
