# Supplementary material for: Functional interactions between NADPH oxidase 5 and actin
Source: Front Cell Dev Biol. 2023 Jan 26;11:1116833. doi: 10.3389/fcell.2023.1116833 (PMC9909703; doi:10.3389/fcell.2023.1116833)
Supplement: Supplementary file 1 [file DataSheet1.PDF]

Supplementary Material

1 Key resources table

Reagent	Source	Identifier
**Antibodies:**		
Anti-NOX5 Rabbit Polyclonal Antibody	Proteintech	Cat# 25350-1-AP, RRID: AB_2811208
Anti-β-Actin Mouse Monoclonal Antibody [AC-15]	Sigma-Aldrich	Cat# A5441, RRID: AB_476744
IRDye 800CW Donkey Anti-Mouse IgG	LI-COR Biosciences	Cat# 926-32212, RRID: AB_621847
IRDye 800CW Goat Anti-Rabbit IgG	LI-COR Biosciences	Cat# 926-32211, RRID: AB-621843
IRDye 680RD Goat Anti-Rabbit IgG	LI-COR Biosciences	Cat# 926-68071, RRID: AB_10956166
IRDye 680RD Donkey Anti-Mouse IgG	LI-COR Biosciences	Cat# 926-68072, RRID: AB_10953628
IRDye 800CW Streptavidin	LI-COR Biosciences	Cat# 926-32230
**Chemicals:**		
L-012	Wako Chemicals	Cat# 120-04891, CAS: 143556-24-5
Cellytic M, Cell Lysis Reagent	Sigma-Aldrich	Cat# C2978
Antibiotic-Antimycotic Solution 100x	Gibco	Cat# 15240062, CAS: 3810-74-0, 69-57-8
Geneticin Selective Antibiotic (G418 Sulfate)	Gibco	Cat# 10131035, CAS: 108321-42-2
Ionomycin	Sigma-Aldrich	Cat# I0634, CAS:56092-82-1
Jasplakinolide	Bio-Techne Sales Corp.	Cat# 2792, CAS: 102396-24-7
Cytochalasin D	Focus Biomolecules	Cat# 10-2071-0001, CAS: 22144-77-0
Cycloheximide	Sigma-Aldrich	Cat# C4859, CAS:66-81-9
Latrunculin A	Focus Biomolecules	Cat# 10-2254, CAS: 76343-93-6
Acetone	Sigma-Aldrich	Cat# 650501, CAS:67-64-1
Tris(2-carboxyethyl)phosphine	Thermo Scientific	Cat# 77712
HEPES	Sigma-Aldrich	Cat# H4034, CAS: 7365-45-9
Maleimide-PEG2-Biotin	Thermo Scientific	Cat# A39261
Streptavidin Magnetic Beads	New England BioLabs	Cat# S1420S
Ammonium Bicarbonate	Sigma-Aldrich	Cat# 09830, CAS: 1066-33-7
N-Ethylmaleimide	Sigma-Aldrich	Cat# E3876, CAS: 128-53-0

EDTA-free Protease Inhibitor Cocktail mini tablet	Sigma-Aldrich	Cat# 11836170001
Protein A-Sepharose 4B	Thermo Scientific	Cat# 101041
Protein G Mag Sepharose	Cytiva	Cat# 28-9940-08
Taqman gene expression assay (NOX5)	Thermo Scientific	Cat# 4331182, Assay ID: Hs00225846_m1
**Commercial Assays:**		
Duolink In Situ Red Starter Kit Mouse/Rabbit	Sigma-Aldrich	Cat# DUO92101
DC Protein Assay Kit II	Bio-Rad	Cat# 5000112
G-Actin/F-Actin In Vivo Assay Biochem Kit	Cytoskeleton	Cat# BK037
FLIPR Calcium 6 Assay Kit	Molecular Devices	Cat# R8194
RNeasy plus micro kit	Qiagen	Cat# 74034
High-capacity cDNA reverse transcription kit	Applied Biosystems	Cat# 4368814
QIAcuity probe kit	Qiagen	Cat# 250102
**Cell Lines:**		
HEK293	ATCC	ATCC# CRL-1573, RRID: CVCL_0045
HEK293, hNOX5, clone B2	Banfi et al, 2001	n/a
PSN-1	ATCC	ATCC# CRL-3211, RRID: CVCL_1644
**Recombinant DNA/siRNA:**		
pcDNA3.1-hNOX5	Banfi et al., 2001	Addgene: 69354
ON-TARGETplus Non-targeting Pool	Dharmacon	Cat# D-001810-10-20
ON-TARGETplus Human NOX5 SMARTpool	Dharmacon	Cat# L-010195-00-0020
**Other:**		
DMEM, high glucose, pyruvate	Sigma-Aldrich	Cat# D7777
DMEM/F-12 15mM HEPES, L-glutamine w/o Phenol Red	Sigma-Aldrich	Cat# D2906
RPMI 1640 Media with L-glutamine	Cytiva	Cat# SH30027.FS
Lipofectamine 2000	ThermoFisher Scientific	Cat# 11668019
TrypLE	Gibco	Cat# 12605010
**Software:**		
Prism	GraphPad	v7, RRID: SCR_002798
Empiria Studio 2.2	LI-COR Biosciences	v2.3.0.154, RRID: SCR_022512
SoftMax Pro Data Acquisition and Analysis Software	Molecular Devices	v7.1.2, RRID: SCR_014240
Cytation C10 Gen5 3.12	BioTek	v3.12, RRID: SCR_017317
QIAcuity software suite	Qiagen	v2.1.7.182

2 Supplementary Figures

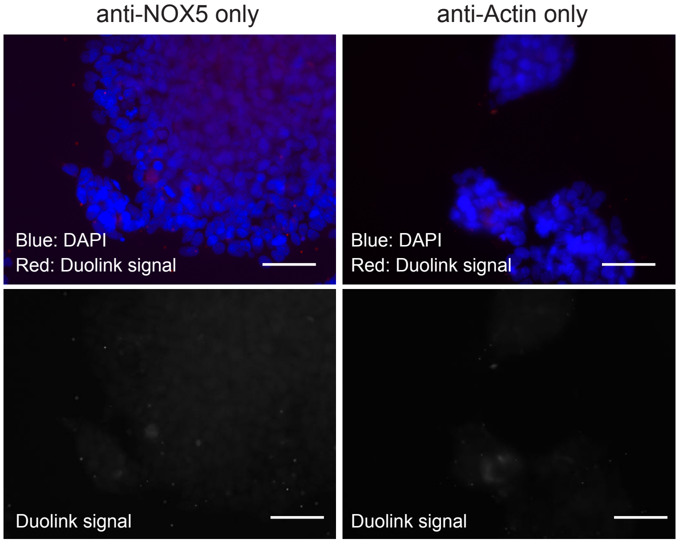

Supplementary Figure 1. Control slides for the Proximity Ligation Assay (PLA) Duolink kit from Sigma with only one primary antibody (either anti-NOX5 or anti-β-actin) but both probes. Top panels show overlap of DAPI (blue) and Duolink signal (red). Bottom panels show red channel signal in greyscale. Addition of only one antibody does not result in red foci. Scale bars are 50 μm.

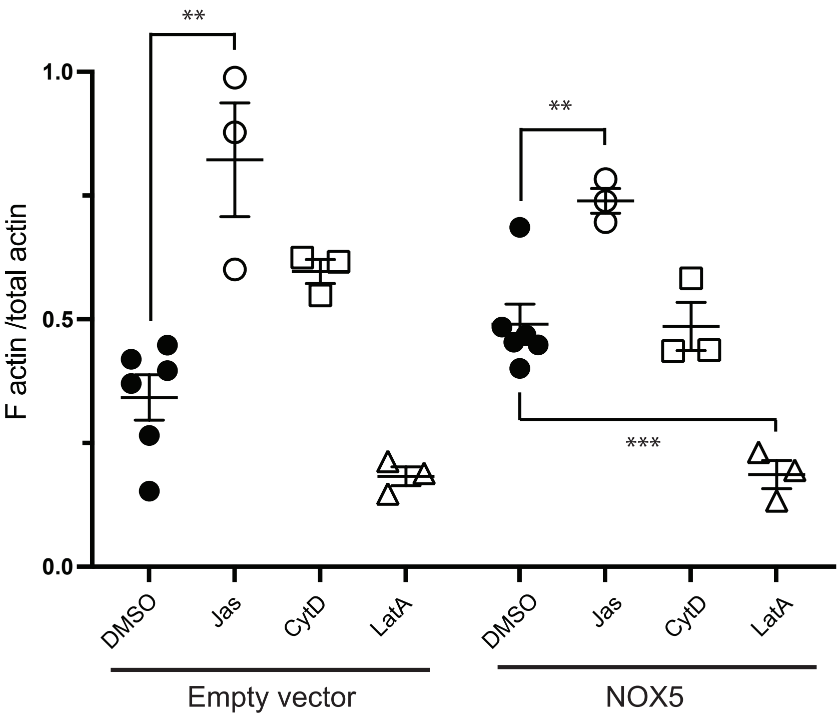

Supplementary Figure 2. Effect of 5 min treatment with 1 μM jasplakinolide (Jas), cytochalasin D (CytD) or latrunculin A (LatA) on the F/G actin ratio in HEK293 cells transfected with empty vector or NOX5. Jasplakinolide increases the amount of F-actin, while latrunculin A decreases F-actin and cytochalasin D has no effect.

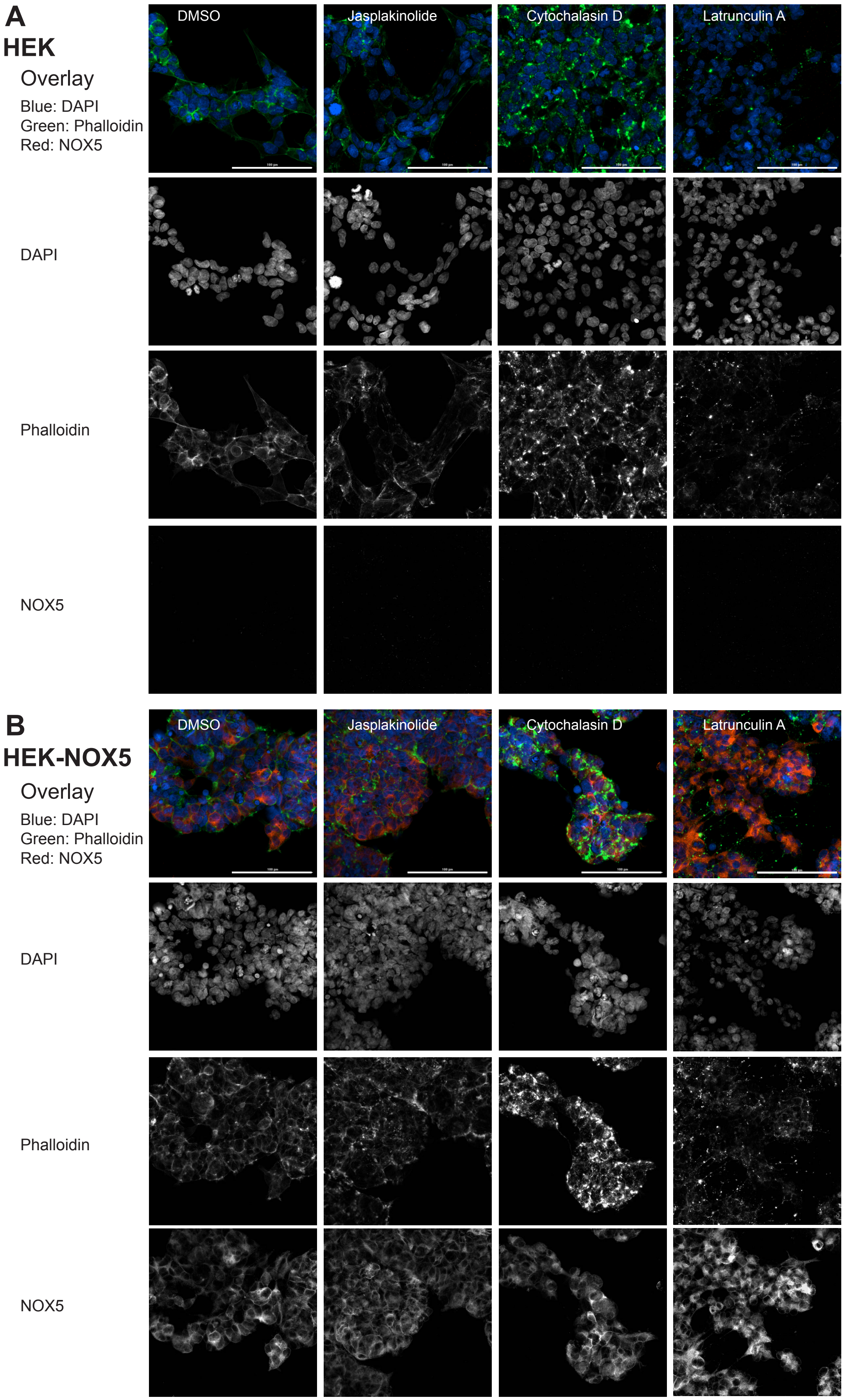
A
HEK
Overlay
Blue: DAPI
Green: Phalloidin
Red: NOX5
DMSO
Jasplakinolide
Cytochalasin D
Latrunculin A
DAPI
Phalloidin
NOX5
B
HEK-NOX5
Overlay
Blue: DAPI
Green: Phalloidin
Red: NOX5
DMSO
Jasplakinolide
Cytochalasin D
Latrunculin A
DAPI
Phalloidin
NOX5

Supplementary Figure 3. Immunofluorescent imaging of HEK293 cells (A) and HEK293 cells stably expressing NOX5 (B) after treatment with actin effectors. Cells were treated with DMSO, jasplakinolide (0.2 µM) cytochalasin D (0.5 µM), or latrunculin A (0.5 µM) for 30 minutes then fixed, permeabilized, probed for NOX5 and stained with phalloidin (filamentous actin) and DAPI (nuclei). Top panels show overlay of DAPI (blue), phalloidin (green) and NOX5 (red). Panels underneath show each in greyscale as labeled. Scale bars are 100 µm.

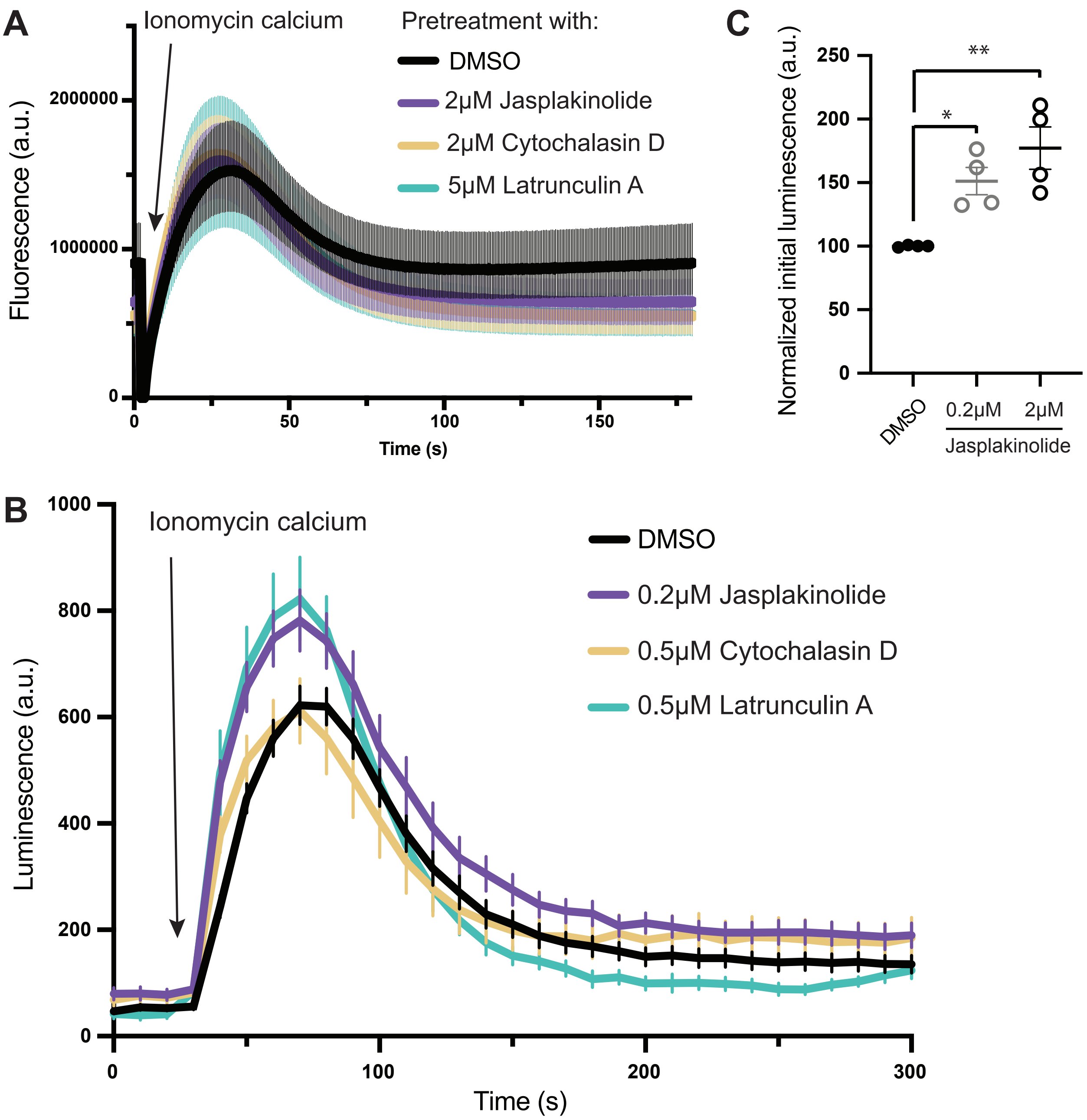

Supplementary Figure 4. (A) Calcium flux after automated addition of ionomycin calcium (1 µM) after pre-treatment for 30 min with jasplakinolide (2 µM), cytochalasin D (2 µM) or latrunculin A (5 µM) measured using the FLIPR Calcium 6 Assay Kit (Molecular Devices) and normalized to fluorescent signal at injection. (B) Timecourse of superoxide production upon addition of ionomycin

calcium salt by HEK293 cells stably expressing NOX5 pre-treated for 30 min with vehicle control (DMSO), jasplakinolide (0.2 µM), cytochalasin D (0.5 µM) or latrunculin A (0.5 µM). (C) Unstimulated superoxide production by NOX5 after treatment for 30 min with DMSO, 0.2 µM or 2 µM jasplakinolide. Values represent means±SEM, n=4. Differences in basal NOX5 activity were assessed using a one-way ANOVA with Tukey's test with * denoting $p < 0.05$ and ** $p < 0.01$.

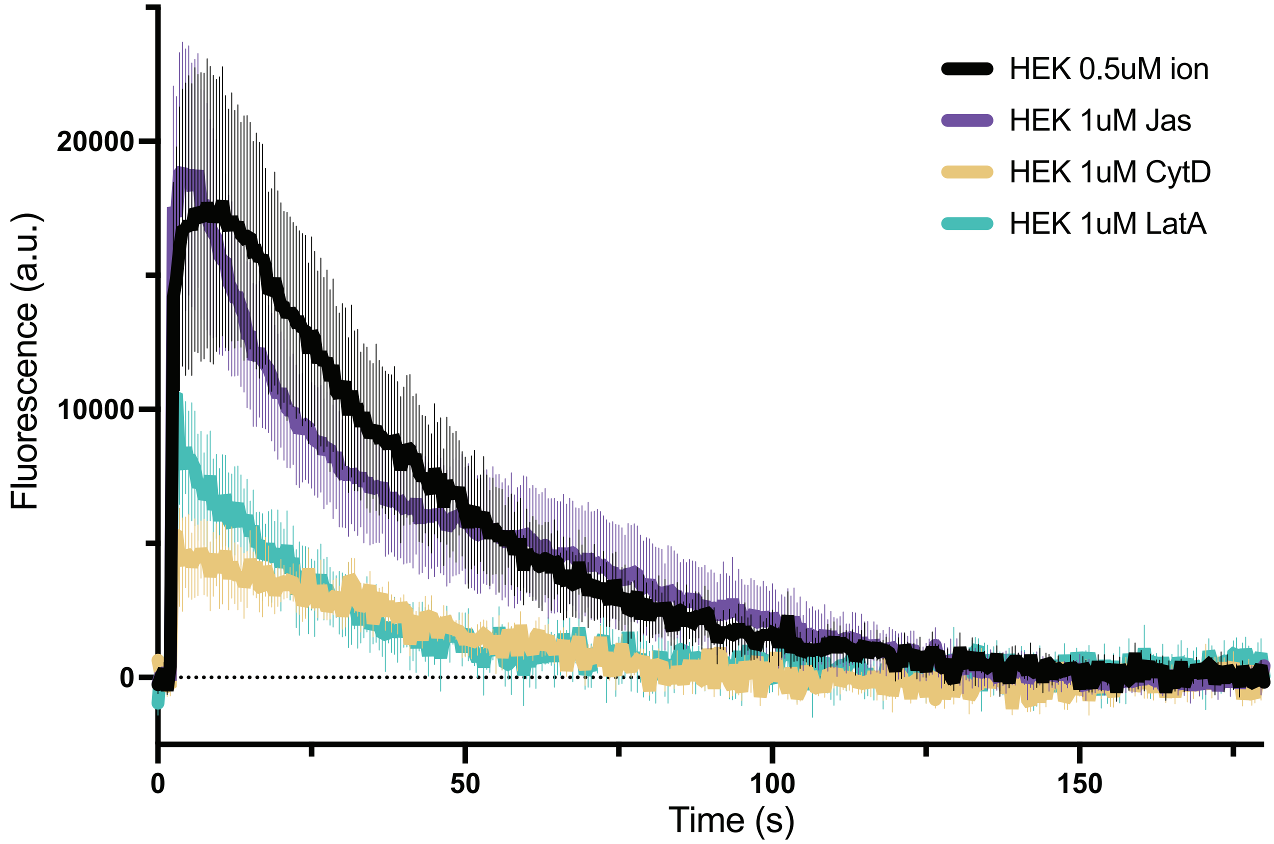

Supplementary Figure 5. Changes in intracellular calcium levels in HEK293 cells after addition of DMSO, ionomycin calcium (0.5 µM), jasplakinolide (1 µM), cytochalasin D (1 µM) or latrunculin A (1 µM) measured using the FLIPR Calcium 6 Assay Kit (Molecular Devices) and normalized to baseline fluorescent signal.

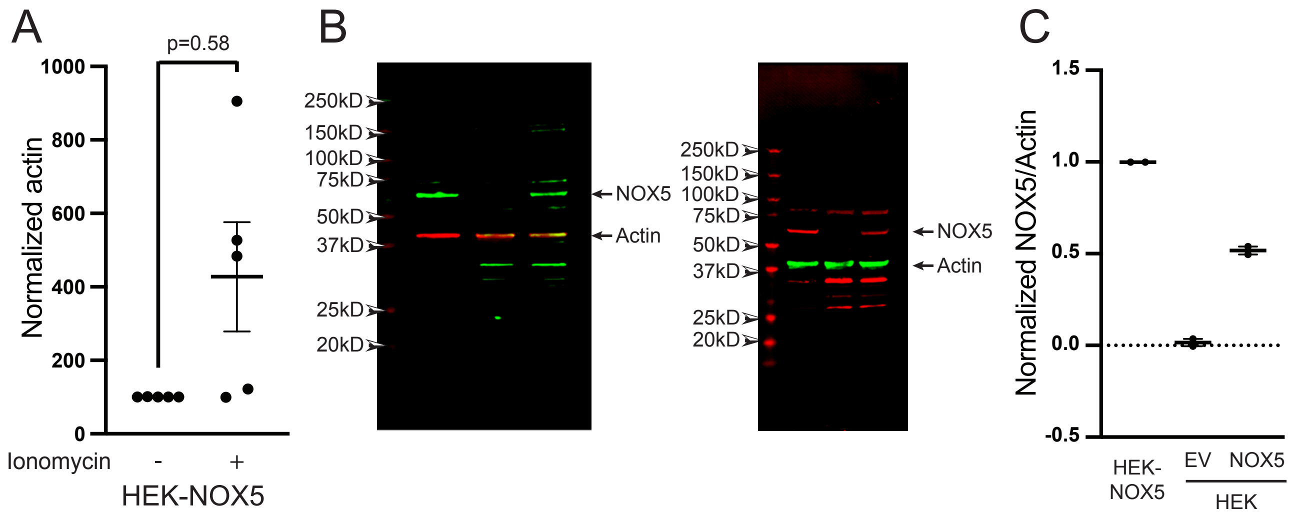

Supplementary Figure 6. (A) Quantification of biotin labeled β-actin in HEK293 cells stably expressing NOX5 treated with DMSO control or 1 μM ionomycin calcium for 5 min. Values represent means±SEM, n=5. Differences in biotin labeled actin between the samples was assessed using a student's t-test with p-value shown on graph. (B) Western blots and quantification (C) of NOX5 and actin in HEK293 cells stably expressing NOX5 and HEK293 cells transiently transfected with empty vector (EV) or NOX5.

Full western blot images

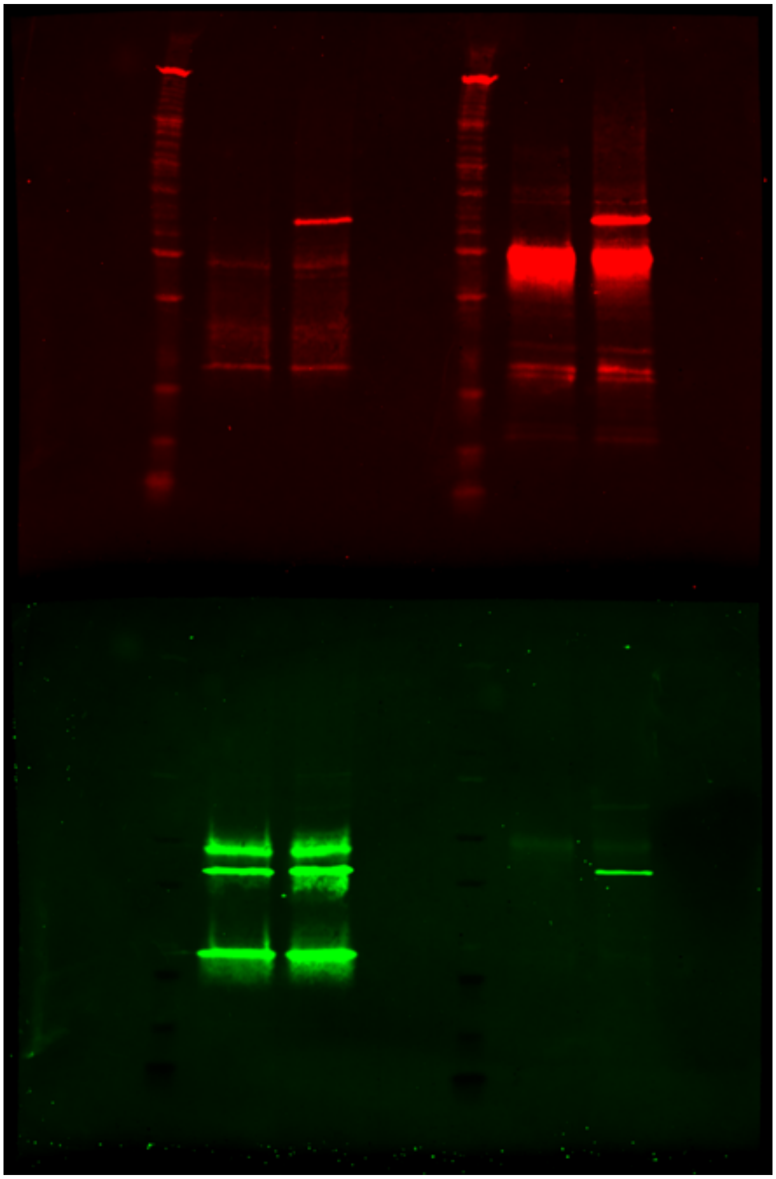

From Figure 1. Top image: IRDye 680RD Goat Anti-Rabbit IgG (For rabbit anti-NOX5) and bottom image: IRDye 800CW Donkey Anti-Mouse IgG (For mouse anti-β-actin). Lanes: 1. Ladder, 2. HEK293 cells IP: actin, 3. HEK293 cells stably expressing NOX5 IP: actin, 4. empty, 5. Ladder, 6. HEK293 cells IP: NOX5, 7. HEK293 cells stably expressing NOX5 IP: NOX5.

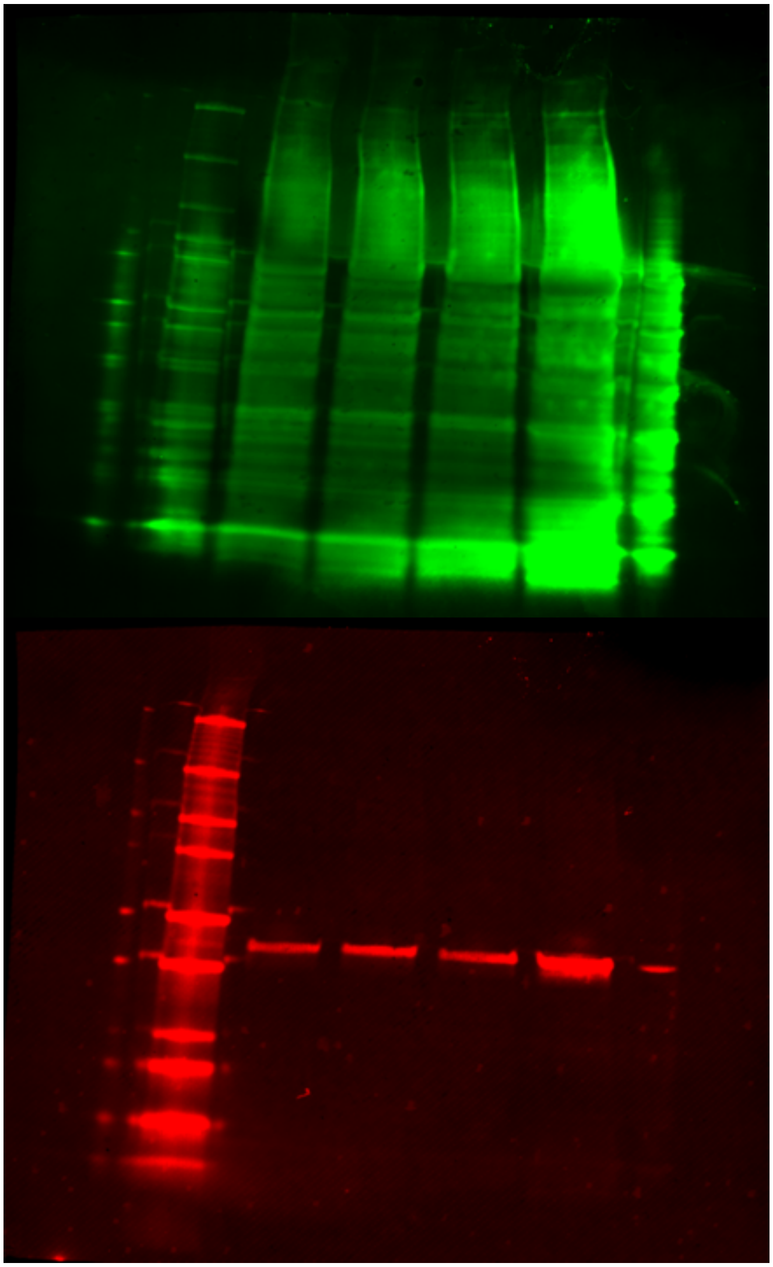

From Figure 4A, C&D. IP: Streptavidin beads. Top image: IRDye 800CW Streptavidin (For biotin) and bottom image: IRDye 680RD Donkey Anti-Mouse IgG (For mouse anti-β-actin). Lanes: 1. Ladder, 2. HEK293 transfected with empty vector + DMSO, 3. HEK293 transfected with empty vector + 1 uM ionomycin calcium, 4. HEK293 transfected with NOX5β + DMSO, 5. HEK293 transfected with NOX5β + 1 uM ionomycin calcium.

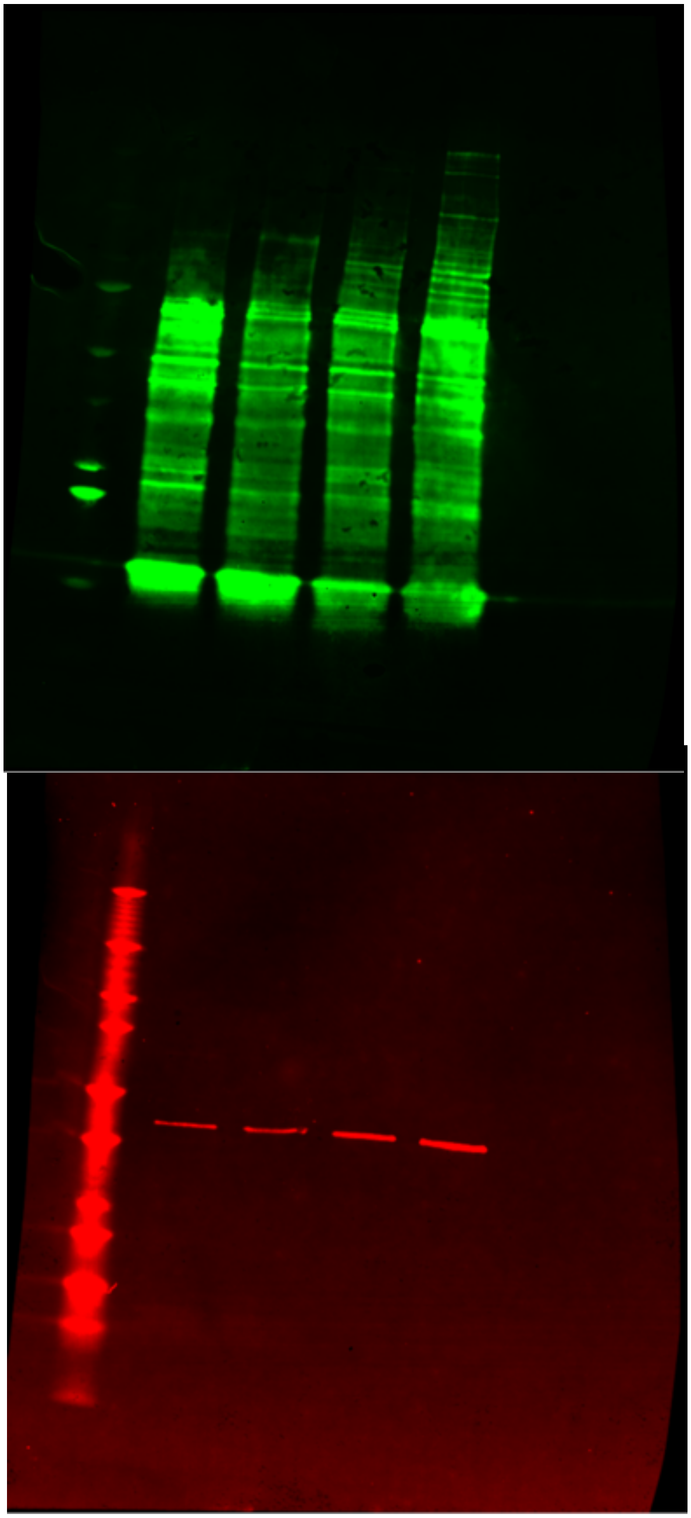

From Figure 4A,D. IP: Streptavidin beads. Top image: IRDye 800CW Streptavidin (For biotin) and bottom image: IRDye 680RD Donkey Anti-Mouse IgG (For mouse anti-β-actin). Lanes: 1. Ladder, 2. HEK293 transfected with empty vector + DMSO, 3. HEK293 transfected with empty vector + 1 uM ionomycin calcium, 4. HEK293 transfected with NOX5β + DMSO, 5. HEK293 transfected with NOX5β + 1 uM ionomycin calcium.

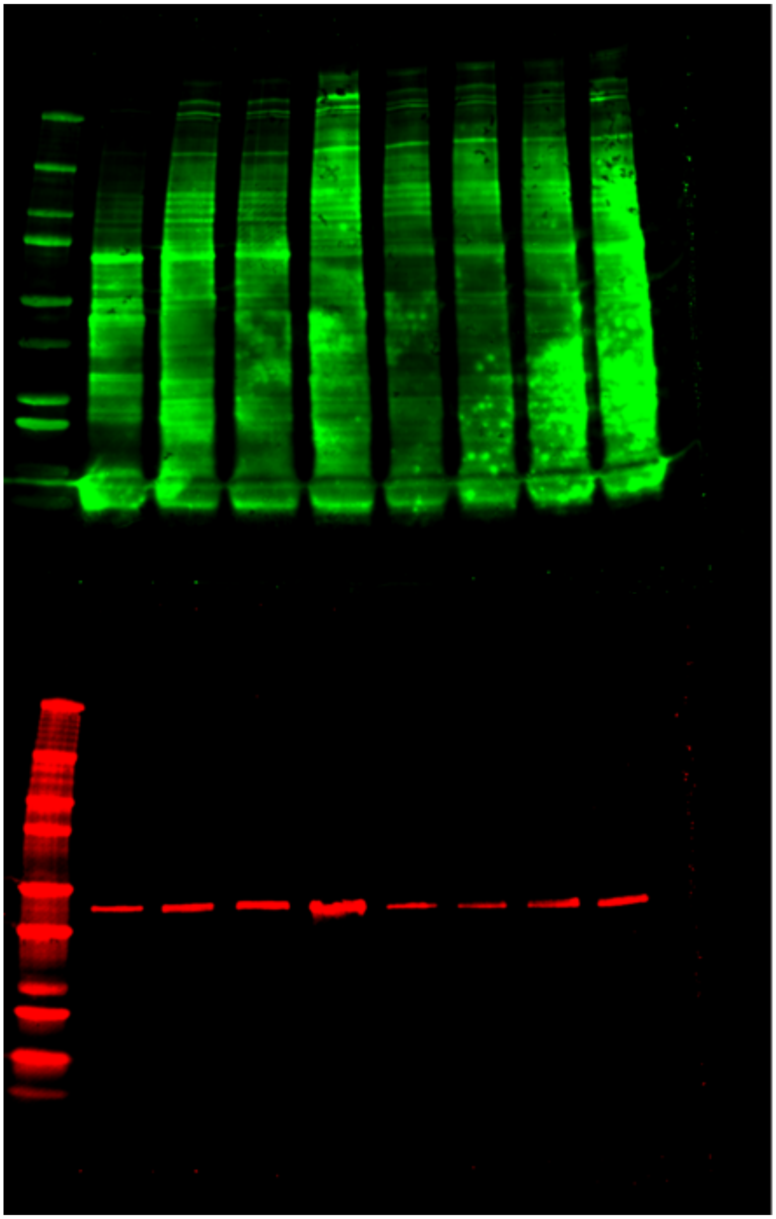

From Figure 4A,B&D. IP: Streptavidin beads, 2 biological replicates. Top image: IRDye 800CW Streptavidin (For biotin) and bottom image: IRDye 680RD Donkey Anti-Mouse IgG (For mouse anti-β-actin). Lanes: 1. Ladder, 2. HEK293 transfected with empty vector + DMSO, 3. HEK293 transfected with empty vector + 1 uM ionomycin calcium, 4. HEK293 transfected with NOX5β + DMSO, 5. HEK293 transfected with NOX5β + 1 uM ionomycin calcium, 6. HEK293 transfected with empty vector + DMSO, 7. HEK293 transfected with empty vector + 1 uM ionomycin calcium, 8. HEK293 transfected with NOX5β + DMSO, 9. HEK293 transfected with NOX5β + 1 uM ionomycin calcium.

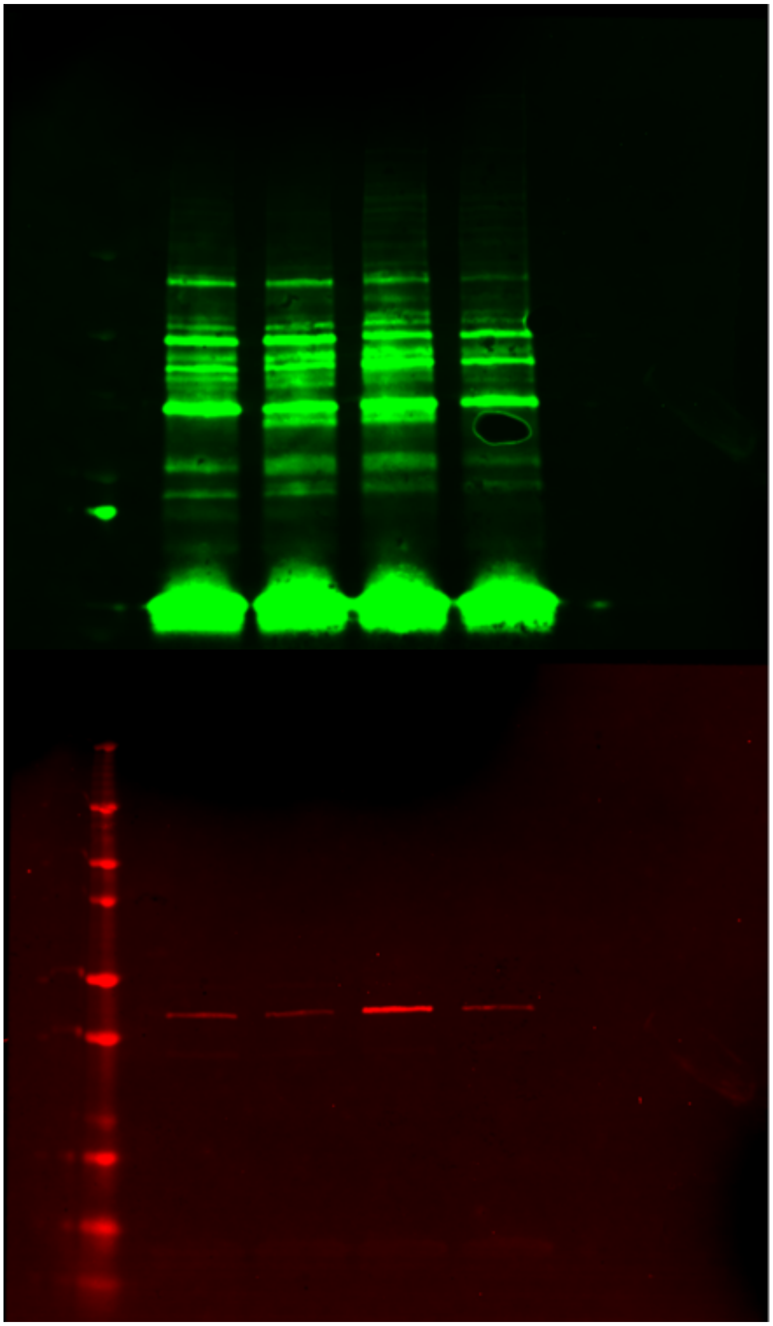

From Figure 4A,D. IP: Streptavidin beads. Top image: IRDye 800CW Streptavidin (For biotin) and bottom image: IRDye 680RD Donkey Anti-Mouse IgG (For mouse anti-β-actin). Lanes: 1. Ladder, 2. HEK293 transfected with empty vector + 1 uM ionomycin calcium, 3. HEK293 transfected with empty vector + DMSO, 4. HEK293 transfected with NOX5β + 1 uM ionomycin calcium, 5. HEK293 transfected with NOX5β + DMSO.

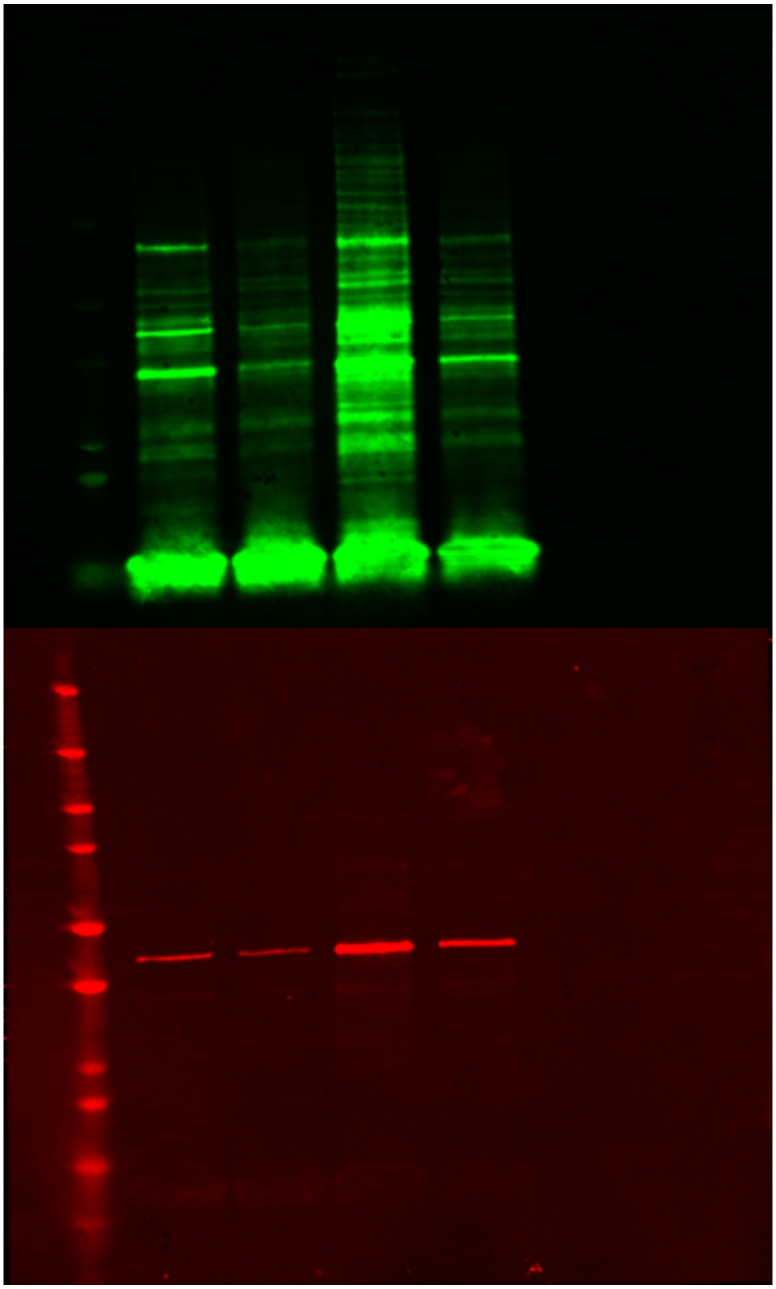

From Figure 4A,D. IP: Streptavidin beads. Top image: IRDye 800CW Streptavidin (For biotin) and bottom image: IRDye 680RD Donkey Anti-Mouse IgG (For mouse anti-β-actin). Lanes: 1. Ladder, 2. HEK293 transfected with empty vector + 1 uM ionomycin calcium, 3. HEK293 transfected with empty vector + DMSO, 4. HEK293 transfected with NOX5β + 1 uM ionomycin calcium, 5. HEK293 transfected with NOX5β + DMSO.

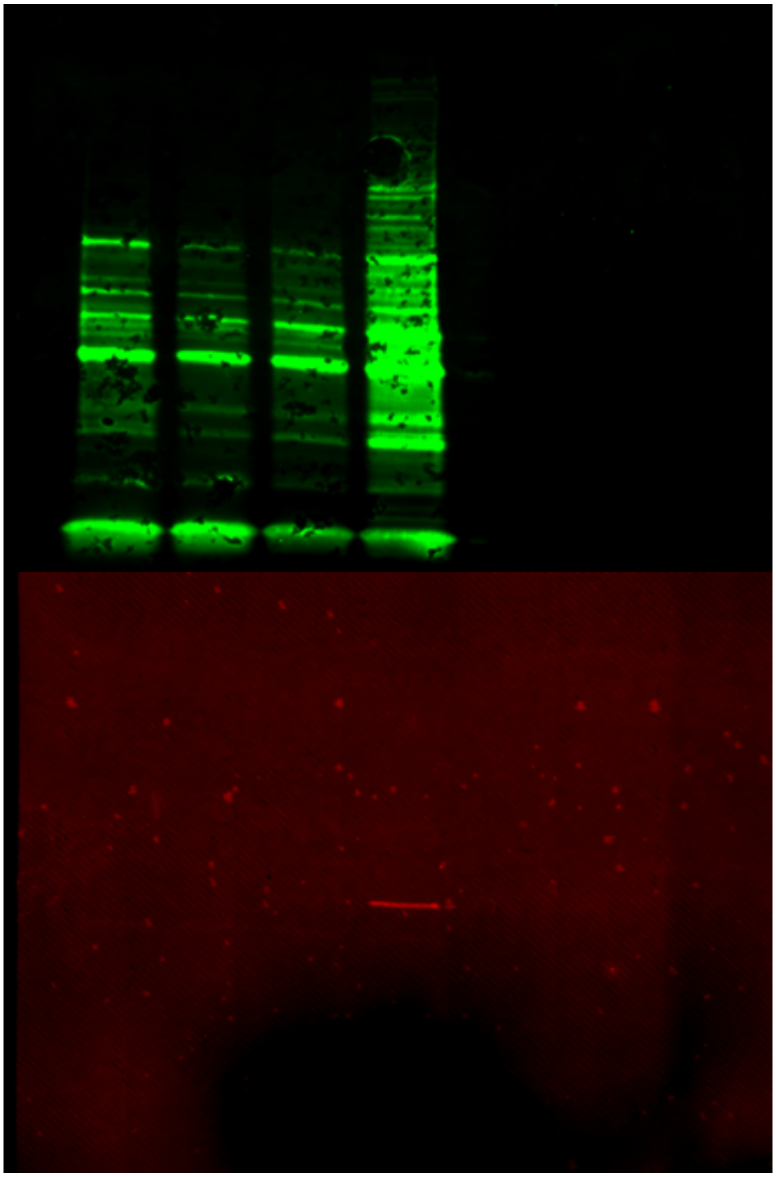

From Figure 4A,D. IP: Streptavidin beads. Top image: IRDye 800CW Streptavidin (For biotin) and bottom image: IRDye 680RD Donkey Anti-Mouse IgG (For mouse anti-β-actin). Lanes: 1. Ladder, 2. HEK293 transfected with empty vector + DMSO, 3. HEK293 transfected with empty vector + 1 uM ionomycin calcium, 4. HEK293 transfected with NOX5β + DMSO, 5. HEK293 transfected with NOX5β + 1 uM ionomycin calcium.

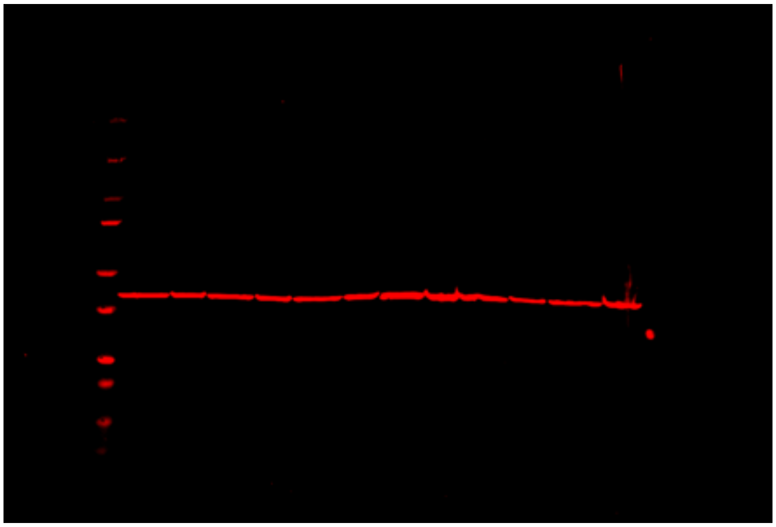

From Figure 4F (lanes used in analysis in bold). IRDye 680RD Donkey Anti-Mouse IgG (For mouse anti-β-actin). Lanes: 1. Ladder, 2. EV none SN, 3. EV none pellet, 4. NOX5 none SN, 5. NOX5 none pellet, **6. EV DMSO SN, 7. EV DMSO pellet, 8. NOX5 DMSO SN, 9. NOX5 DMSO pellet**, 10. EV ion SN, 11. EV ion pellet, 12. NOX5 ion SN, 13. NOX5 ion pellet

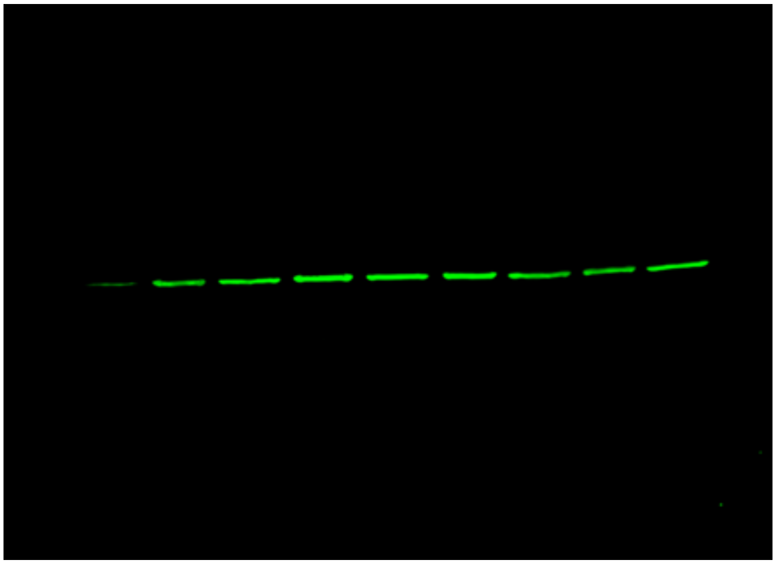

From Figure 4F (lanes used in analysis in bold). IRDye 800CW Donkey Anti-Mouse IgG (For mouse anti-β-actin). 1. ladder, 2. *loading error, skip, **3. EV DMSO** pellet, 4. EV ion SN, 5. EV ion pellet, **6. NOX5 DMSO SN, 7. NOX5 DMSO pellet**, 8. NOX5 ion SN, 9. NOX5 ion pellet, **10. #2 - EV DMSO SN**

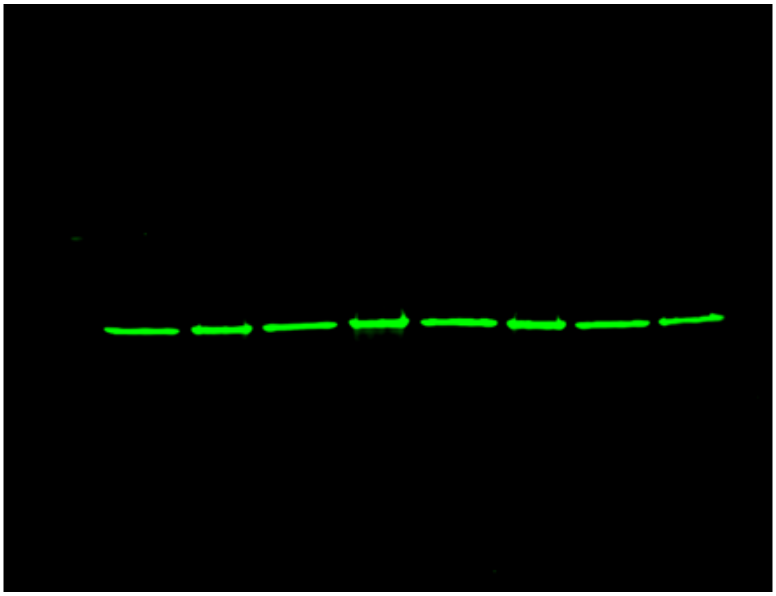

From Figure 4F (lanes used in analysis in bold). IRDye 800CW Donkey Anti-Mouse IgG (For mouse anti-β-actin). 1. ladder, **2. EV DMSO SN, 3. EV DMSO pellet,** 4. EV ion SN, 5. EV ion pellet, **6. NOX5 DMSO SN, 7. NOX5 DMSO pellet,** 8. NOX5 ion SN, 9. NOX5 ion pellet.

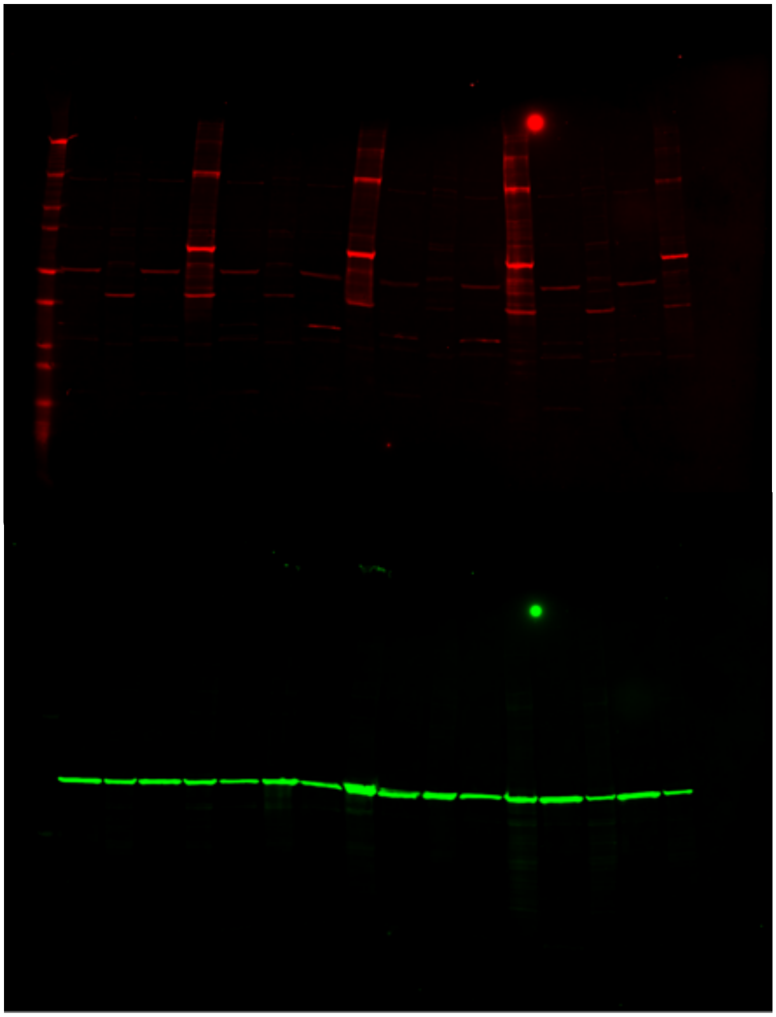

From Figure 4E, F, Supplementary Figure 2. Top image: IRDye 680RD Goat Anti-Rabbit IgG (For rabbit anti-NOX5) and bottom image: IRDye 800CW Donkey Anti-Mouse IgG (For mouse anti-β-actin). Lanes: 1. Ladder, 2. EV DMSO SN, 3. EV DMSO pellet, 4. NOX5 DMSO SN, 5. NOX5 DMSO pellet, 6. EV Jasp SN, 7. EV jasp pellet, 8. NOX5 jasp SN, 9. NOX5 jasp pellet, 10. EV cytD SN, 11. EV cytD pellet, 12. NOX5 cytD SN, 13. NOX5 cytD pellet, 14. EV latA SN, 15. EV lat A pellet, 16. NOX5 latA SN, 17. NOX5 latA pellet.

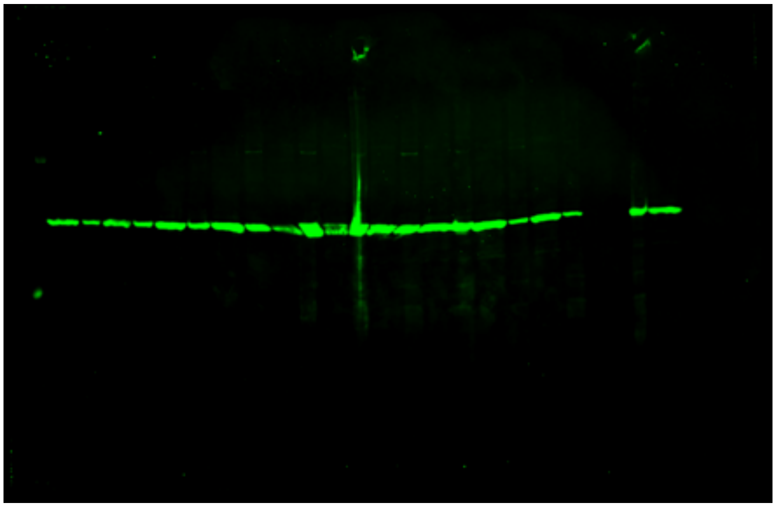

From Figure 4F, Supplementary Figure 2. IRDye 800CW Donkey Anti-Mouse IgG (For mouse anti-β-actin). Lanes 8 and 13 repeated at the end. 1. Ladder, 2. EV dmso sn, 3. EV dmso pellet, 4. nox5 dmso sn, 5. nox5 dmso pellet, 6. EV ion sn, 7. ev ion pellet, 8. nox5 ion sn, 9. nox5 ion pellet, 10. ev jasp sn, 11. ev jasp pellet 12. nox5 jasp sn, 13. nox5 jasp pellet, 14. ev cytd sn, 15. ev cytd pellet, 16. nox5 cytd sn, 17. nox5 cytd pellet, 18. ev lat a sn, 19. ev lat a pellet, 20. nox5 lat a sn, 21. nox5 lat a pellet, 22. Empty, 23. Rerun of #13 (nox5 jasp pellet), 23. Rerun of #8 (nox5 ion sn)

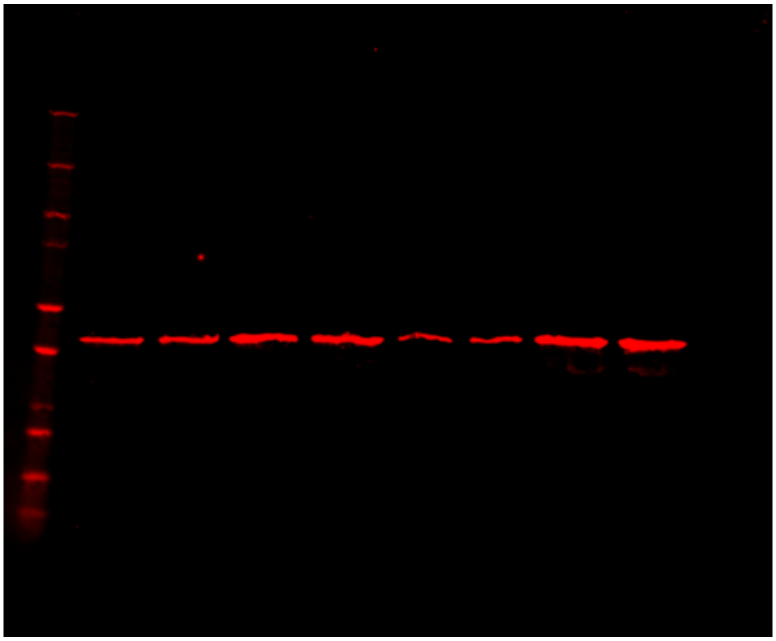

From Figure 4H (lanes used in analysis in bold). IRDye 680RD Donkey Anti-Mouse IgG (For mouse anti-β-actin). Lanes: 1. Ladder, **2. HEK DMSO pellet,** 3. HEK pellet cyclo, **4. HEK DMSO SN**, 5. HEK SN cyclo, **6. B2 DMSO pellet**, 7. B2 pellet cyclo, **8. B2 DMSO SN**, 9. B2 SN cyclo.

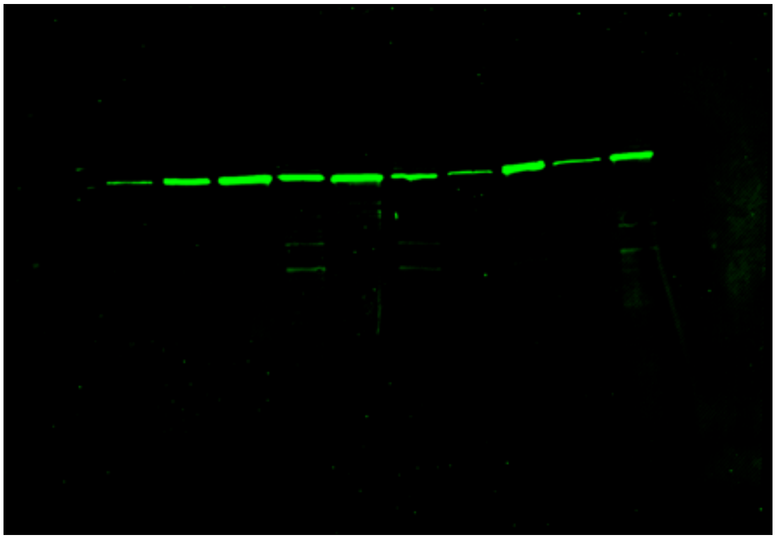

From Figure 4H (lanes used in analysis in bold). IRDye 800CW Donkey Anti-Mouse IgG (For mouse anti-β-actin). Lanes: Ladder, 1. Actin STD 10, 2. Actin STD 20, 3. Actin STD 50, **4. HEK SN DMSO, 5. HEK pellet DMSO, 6. B2 SN DMSO, 7. B2 pellet DMSO**, 8. HEK SN Jasp 30 min, 9. HEK pellet Jasp 30 min, 10. B2 SN Jasp 30 min,11. B2 pellet Jasp 30 min

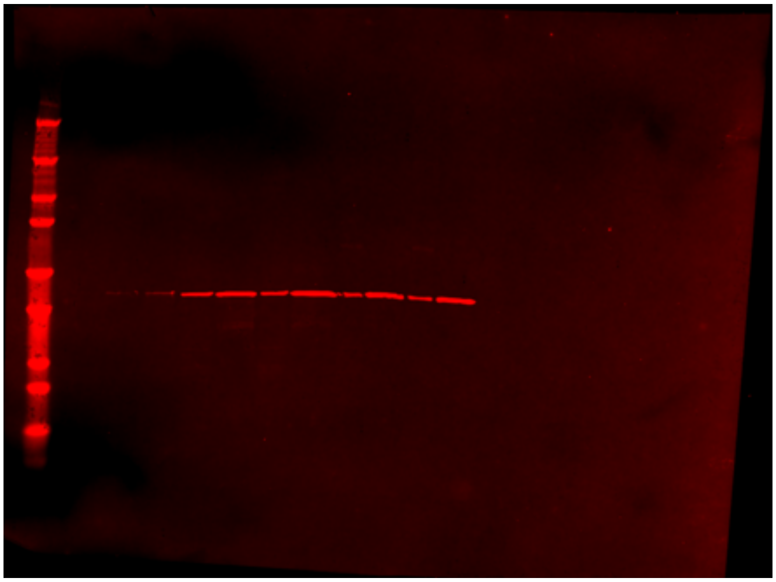

From Figure 4H. (lanes used in analysis in bold). IRDye 680RD Donkey Anti-Mouse IgG (For mouse anti-β-actin). Lanes: 1. Ladder, 2. 20 STD, 3. 40 STD, 4. 100 STD, 5. HEK pellet, 6. HEK SN, **7. HEK DMSO pellet, 8. HEK DMSO SN,** 9. B2 pellet, 10. B2 SN, **11. B2 DMSO pellet, 12. B2 DMSO SN.**

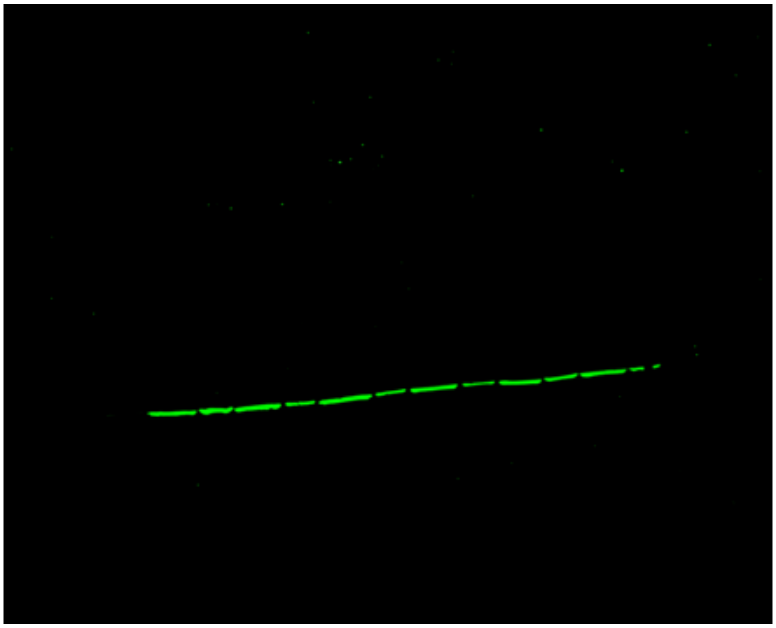

From Figure 4H. (lanes used in analysis in bold). IRDye 800CW Donkey Anti-Mouse IgG (For mouse anti-β-actin). Lanes: 1. Ladder, 2. 120 STD, 3. 180 STD, 4. HEK no treatment SN, 5. HEK no treatment pellet, 6. B2 no treatment SN, 7. B2 no treatment pellet, **8. HEK DMSO SN, 9. HEK DMSO pellet, 10. B2 DMSO SN, 11. B2 DMSO pellet,** 12. HEK ionomycin SN, 13. HEK ionomycin pellet, 14. B2 ionomycin SN, 15. B2 ionomycin pellet.

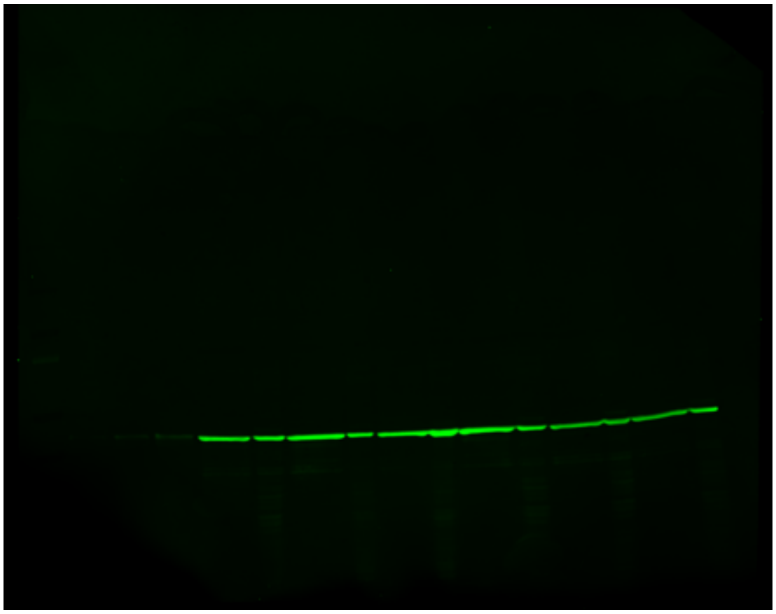

From Figure 4H. (lanes used in analysis in bold). IRDye 800CW Donkey Anti-Mouse IgG (For mouse anti-β-actin). Lanes: 1. Ladder, 2. 60 STD, 3. 120 STD, 4. 180 STD, 5. HEK no treatment SN, 6. HEK no treatment pellet, 7. B2 no treatment SN, 8. B2 no treatment pellet, **9. HEK DMSO SN, 10. HEK DMSO pellet, 11. B2 DMSO SN, 12. B2 DMSO pellet,** 13. HEK ionomycin SN, 14. HEK ionomycin pellet, 15. B2 ionomycin SN, 16. B2 ionomycin pellet.

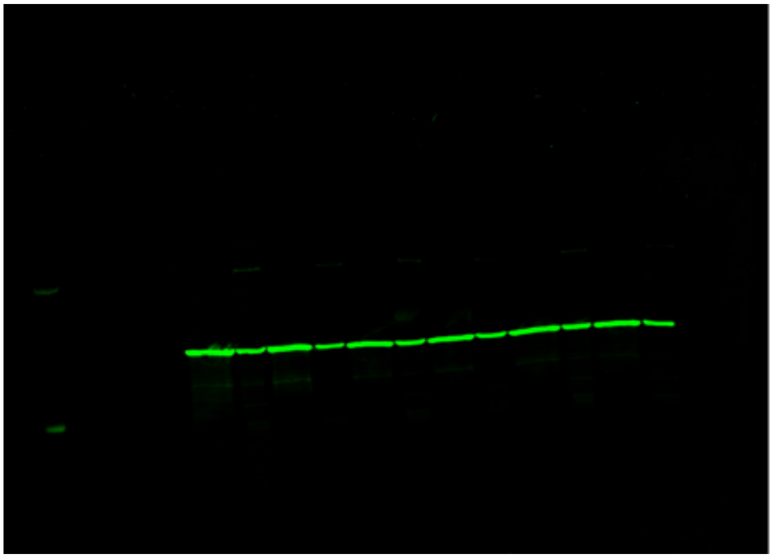

From Figure 4H. (lanes used in analysis in bold). IRDye 800CW Donkey Anti-Mouse IgG (For mouse anti-β-actin). Lanes: 1. Ladder, 2. 60 STD, 3. 120 STD, 4. 180 STD, 5. HEK no treatment SN, 6. HEK no treatment pellet, 7. B2 no treatment SN, 8. B2 no treatment pellet **9. HEK DMSO SN, 10. HEK DMSO pellet, 11. B2 DMSO SN, 12. B2 DMSO pellet,** 13. HEK ion SN, 14. HEK ion pellet, 15. B2 ion SN, 16. B2 ion pellet.

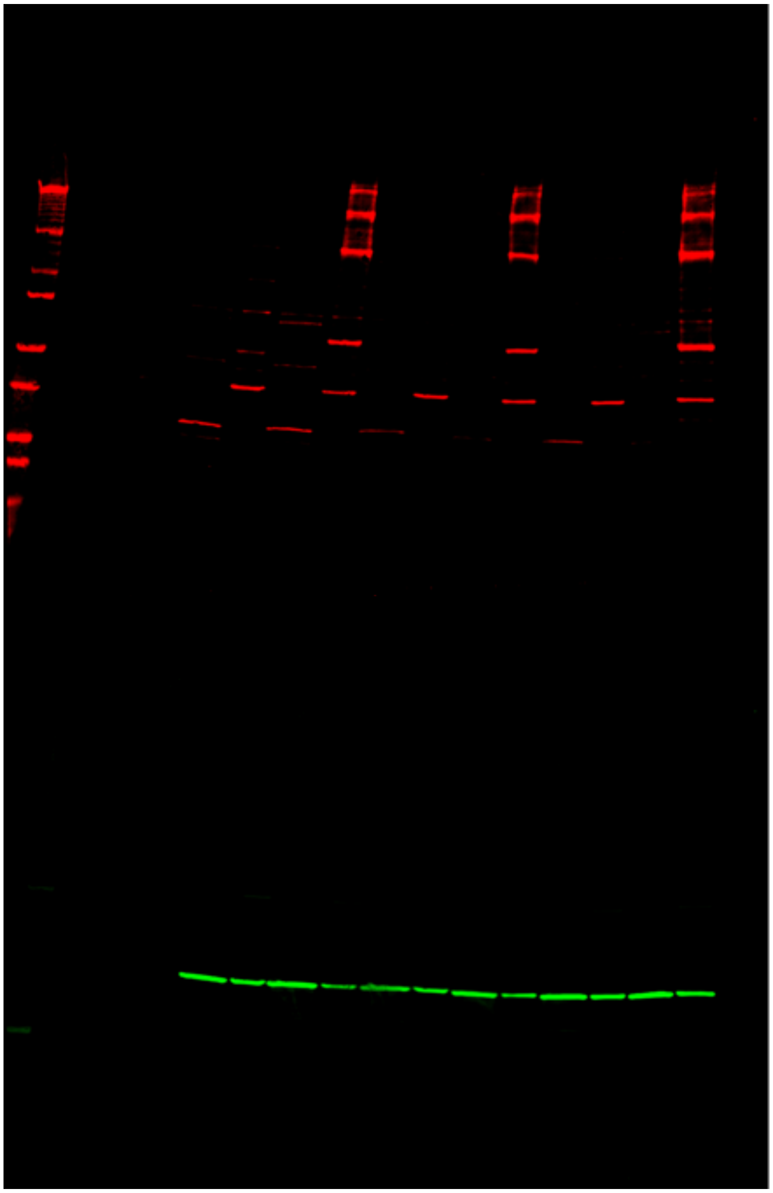

From Figure 4G&H. (lanes used in analysis in bold). Top image: IRDye 680RD Goat Anti-Rabbit IgG (For rabbit anti-NOX5) and bottom image: IRDye 800CW Donkey Anti-Mouse IgG (For mouse anti-β-actin). Lanes: 1. Ladder, 2. 60 STD, 3. 120 STD, 4. 180 STD, 5. HEK no treatment SN, 6. HEK no treatment pellet, 7. B2 no treatment SN, 8. B2 no treatment pellet **9. HEK DMSO SN, 10. HEK DMSO pellet, 11. B2 DMSO SN, 12. B2 DMSO pellet,** 13. HEK ion SN, 14. HEK ion pellet, 15. B2 ion SN, 16. B2 ion pellet.

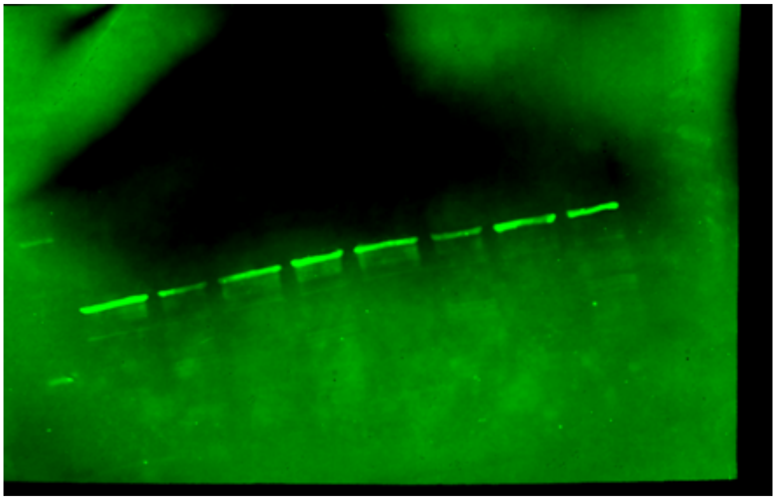

From Figure 4F. (lanes used in analysis in bold). IRDye 800CW Donkey Anti-Mouse IgG (For mouse anti-β-actin). Lanes: 1. Ladder, **2. ev dmso sn, 3. ev dmso pellet, 4. nox5 dmso sn, 5. nox5 dmso pellet,** 6. ev ion sn, 7. ev ion pellet, 8. nox5 ion sn, 9. nox5 ion pellet.

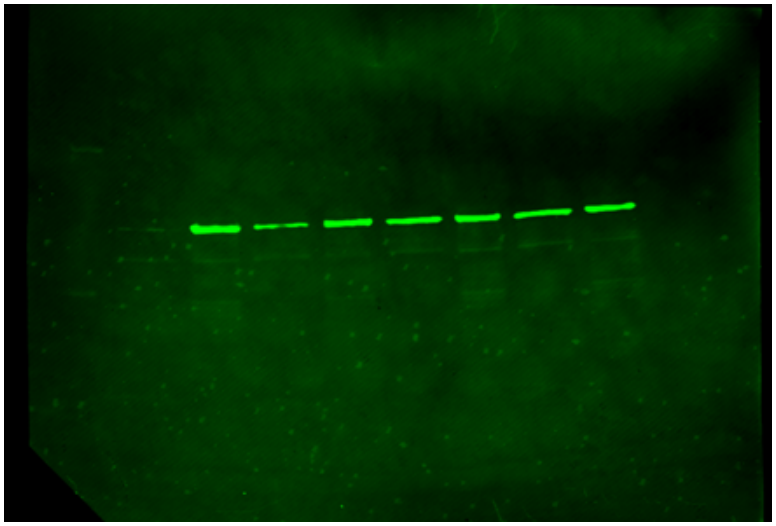

From Supplementary Figure 2. IRDye 800CW Donkey Anti-Mouse IgG (For mouse anti-β-actin). Lanes: 1. Ladder, 2. ev jasp sn, 3. ev jasp pellet, 4. nox5 jasp sn, 5. nox5 jasppellet, 6. ev CytD sn, 7. ev CytD pellet, 8. nox5 CytD sn, 9. nox5 CytD pellet

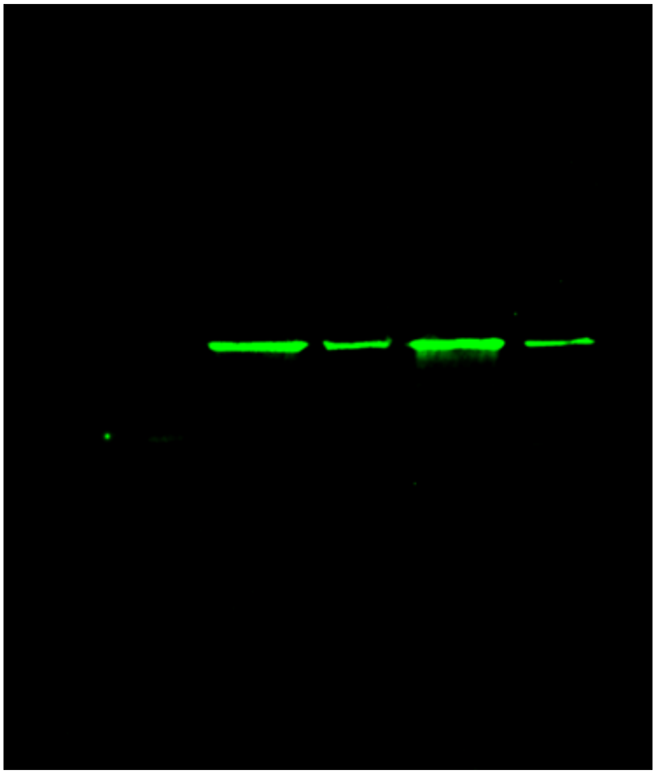

From Supplementary Figure 2. IRDye 800CW Donkey Anti-Mouse IgG (For mouse anti-β-actin). Lanes: 1. Ladder, 2. ev latA sn, 3. ev latA pellet, 4. nox5 latA sn, 5. nox5 latA pellet.
